# Estimating Location without External Cues

**DOI:** 10.1371/journal.pcbi.1003927

**Published:** 2014-10-30

**Authors:** Allen Cheung

**Affiliations:** The University of Queensland, Queensland Brain Institute, Brisbane, Queensland, Australia; Indiana University, United States of America

## Abstract

The ability to determine one's location is fundamental to spatial navigation. Here, it is shown that localization is theoretically possible without the use of external cues, and without knowledge of initial position or orientation. With only error-prone self-motion estimates as input, a fully disoriented agent can, in principle, determine its location in familiar spaces with 1-fold rotational symmetry. Surprisingly, localization does not require the sensing of any external cue, including the boundary. The combination of self-motion estimates and an internal map of the arena provide enough information for localization. This stands in conflict with the supposition that 2D arenas are analogous to open fields. Using a rodent error model, it is shown that the localization performance which can be achieved is enough to initiate and maintain stable firing patterns like those of grid cells, starting from full disorientation. Successful localization was achieved when the rotational asymmetry was due to the external boundary, an interior barrier or a void space within an arena. Optimal localization performance was found to depend on arena shape, arena size, local and global rotational asymmetry, and the structure of the path taken during localization. Since allothetic cues including visual and boundary contact cues were not present, localization necessarily relied on the fusion of idiothetic self-motion cues and memory of the boundary. Implications for spatial navigation mechanisms are discussed, including possible relationships with place field overdispersion and hippocampal reverse replay. Based on these results, experiments are suggested to identify if and where information fusion occurs in the mammalian spatial memory system.

## Author Summary

Spatial navigation is one of the most important functions of animal brains. Multiple regions and cell types encode the current location in mammalian brains, but the underlying interactions between sensory and memory information remain unclear. Recent experimental and theoretical evidence have been found to suggest that the presence of a boundary fundamentally alters the task of navigation. In this paper, evidence is provided that it is possible to determine the location inside any familiar arena with 1-fold rotational symmetry, while completely ignoring sensory cues from the outside world. Surprisingly, the results show that the mere knowledge of the boundary's existence is enough, without requiring direct physical contact. Localization is robust despite the presence of noise modelled from the rodent head direction system, and even inaccuracies in the navigation system's memory of the boundary or internal models of noise. In circular arenas, rotational asymmetry can arise from interior structures such as barriers or voids, also without contact information. This theoretical evidence highlights the need to distinguish arena-based navigation common to most experimental studies, from open field navigation. These findings also point to novel ways to study information fusion in mammalian brains.

## Introduction

Accurate spatial navigation is crucial to animal survival. Localization is the process of determining current location, critical for many navigation behaviours. Starting from an unknown location and direction (jointly called “pose”), the ability to localize is thought to depend on the detection of world-based (‘allothetic’) cues such as visual landmarks. In contrast, it is thought that animal-based (‘idiothetic’) cues which provide self-motion estimates, e.g., from vestibular, proprioceptive or motor command signals, can only serve to maintain localization briefly, requiring allothetic cues for error correction [1]–[5]. This is because cumulative errors will degrade self-motion estimates of position over time, not improve it.

In the rodent brain, the firing of both place and grid cells strongly correlate with the animal's physical location in a familiar space [6]–[10], with firing patterns being stable over days to weeks in the same environment [11]. Such neural correlates demonstrate that an animal can robustly localize itself within a familiar arena. A consistent feature of these neural correlates of localization is their persistence without vision, for upwards of 30 minutes [12], [13]. One possible strategy is to use idiothetic path integration (iPI) whereby an animal keeps track of its current position by summing idiothetic estimates of displacement [8], [14]–[16], but this process is known to suffer from cumulative errors. In an open field, error accumulation due to iPI will lead to a rapid increase in discrepancy between true position and estimated position [14]–[16]. It follows that, if using only iPI, a navigation system cannot accurately estimate its location in the long term, and certainly cannot localize itself starting from an unknown pose. An important biological implication is that stable, spatially-selective firing patterns such as those of rodent hippocampal place cells or medial entorhinal grid cells cannot depend purely on iPI.

Most experiments investigating neural correlates of spatial behaviour have been performed in either linear tracks or 2D arenas, the latter termed ‘open fields’ [9], [10]. On their own, featureless arena boundaries do not provide sufficient spatial information for localization without vision. This is due to a combination of geometric properties [17], and infinite poses which equally account for the detection of a point along a featureless boundary during boundary contact. The problem is compounded further if boundary contact (an allothetic cue) is not available or used. The mere knowledge of a boundary's geometry is therefore insufficient for localization, and might be interpreted as support that arena boundaries do not significantly aid localization compared to boundary-less open fields.

Contrary to the above supposition, it is demonstrated here that long-term accurate localization is possible if idiothetic self-motion cues are combined with boundary information already in memory. In particular, the previously acquired boundary map limits the growth of uncertainty due to noisy self-motion information. Surprisingly, the act of sensing the boundary or any other landmark is not necessary. Metrics based on information theoretic principles are used to quantify localization performance. Grid cell simulations are used to provide both a visual display of time-averaged localization performance, and to predict the optimal spatial selectivity that may be expected of neural firing patterns, based on published rodent neural data.

## Results

### PI alone cannot maintain an accurate position estimate in the long term, even with a compass

**Figure 1:**
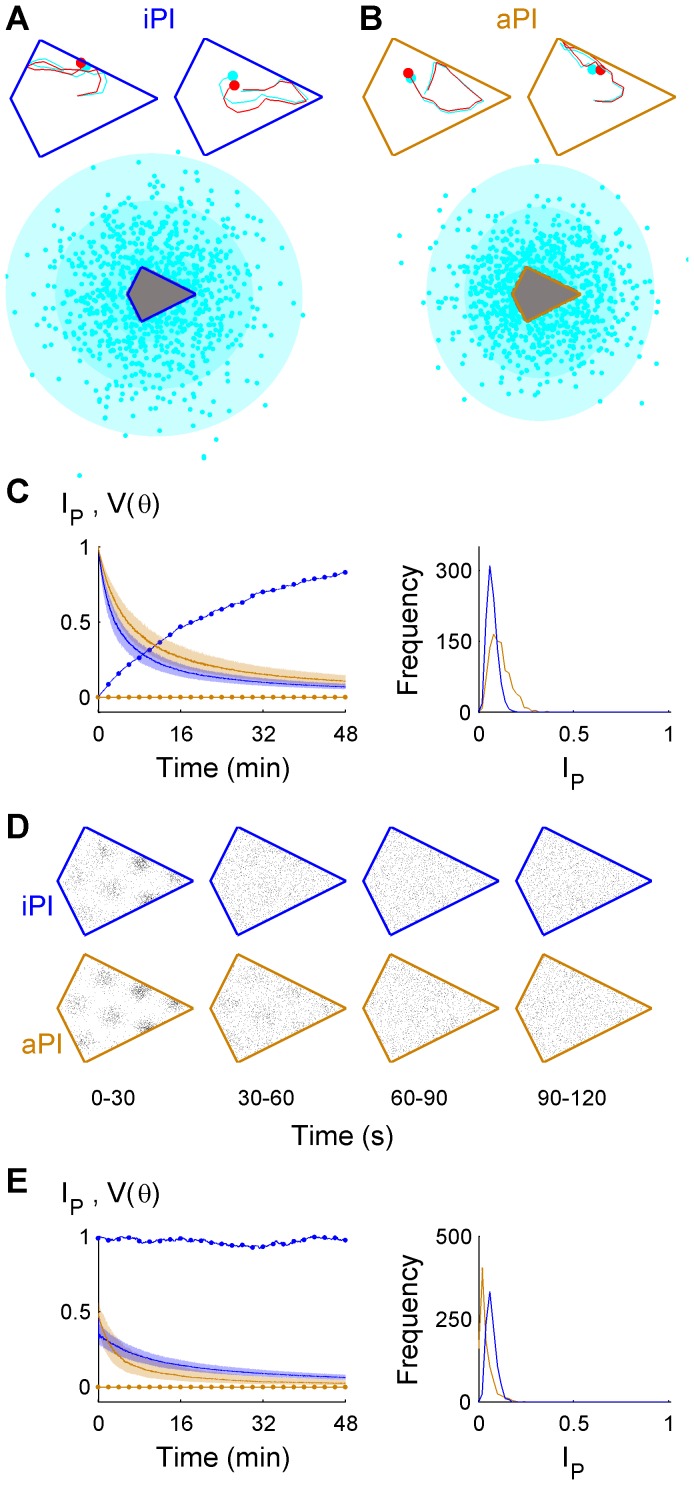
Failure of localization using only path integration. Examples of the first 20 true steps along random trajectories (red), using iPI only (**A**) or aPI only (**B**) to track the position (cyan), starting at the arena centre fully oriented. The estimated final position (cyan, with bivariate Gaussian fit showing 1, 2 and 3 SDs) following 48 minutes for 10^3^ independent trials differed markedly from the true positions (red, approximately uniformly random within the arena). (**C**) Initially oriented, *I_p_* (solid lines, median; shaded, IQR) decayed more rapidly using iPI (blue, 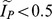 by 3:07) than aPI (orange, 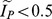 by 5:33). *V(θ)* increased using iPI (blue dotted line), but *V(θ)* remained constant during aPI (orange dotted line). The *I_p_* distributions (right) showed that all position estimates were below chance (0.5) at 48 minutes. (**D**) Simulated grid cell spikes using iPI (top row) and aPI (bottom row) had insufficient spike count (averaging <1 spike per analysis pixel) to obtain reliable measures of information content. (**E**) Initially disoriented, 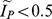 throughout the 48 minute period (lower than (**C**), Wilcoxon test: iPI, p = 1.4×10^−4^; aPI, p = 3.3×10^−5^), and no grid firing pattern formed. Stable *V(θ)* showed no gain or loss of directional information.

As a baseline, both iPI and aPI (allothetic PI implies PI using a compass) performance were quantified without arena memory, in a kite-shaped arena. Even for the simplified task of PI initially orientated, localization failed (Fig. 1A, 1B). The median place stability index, 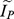, fell below 0.5 (chance level) in 3 minutes using iPI alone, and under 6 minutes using aPI alone (Fig. 1C), consistent with the generally-accepted idea that cumulative PI errors degrade location estimates over time. Using iPI, the circular variance 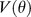 which measures the particle filter's directional performance across trials, increased from 0 (no error) to close to 1 (uniformly random orientation). In contrast, 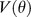 remained close to 0 using aPI since the compass continually reset orientation errors. Using either type of PI, spatially-selective firing patterns could not be maintained beyond 1–2 minutes (Fig. 1D). The more general task of localization initially disoriented (Fig. 1E) further increased localization difficulty, with 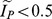 throughout the simulation period, and no grid-like firing pattern was observed. Taken together, it was clear that neither aPI or iPI alone could enable localization. Next, idiothetic self-motion cues were combined with arena memory information.

### Idiothetic localization inside a 2D arena

**Figure 2:**
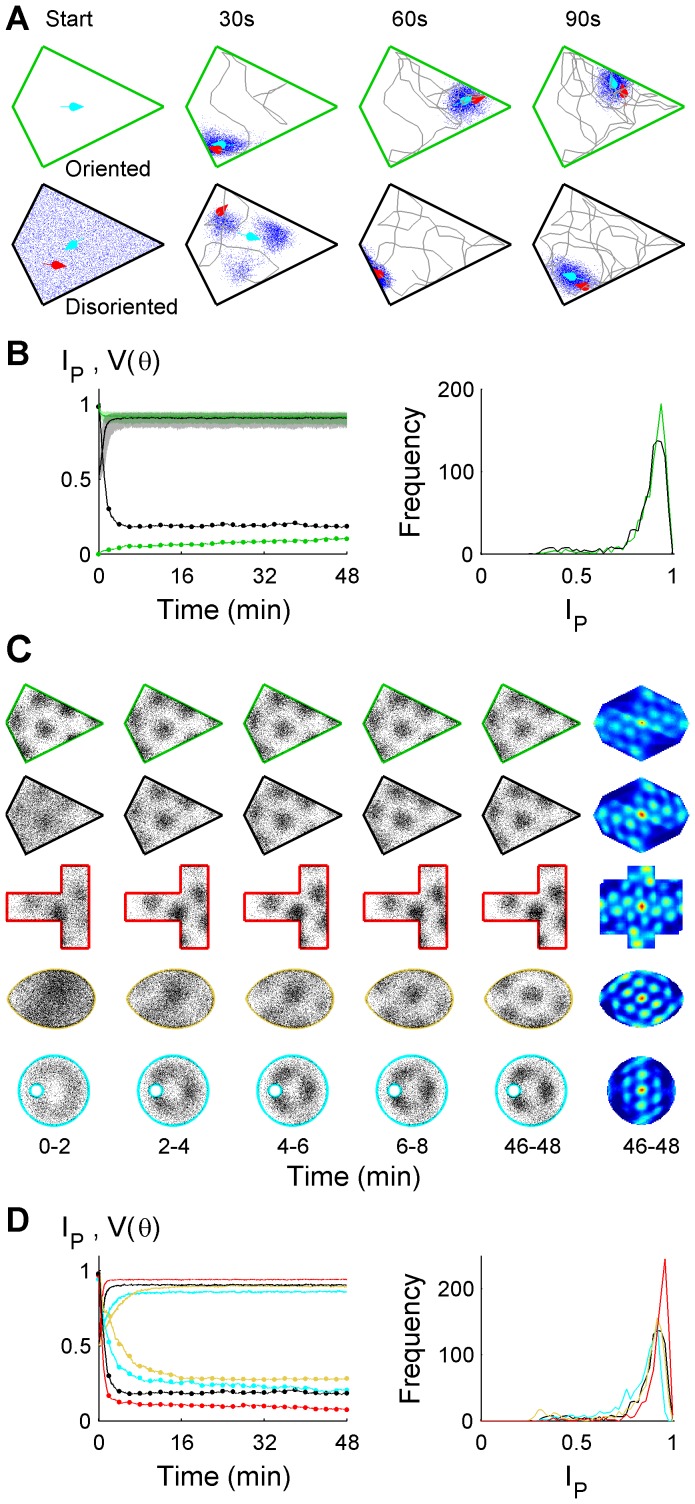
Successful idiothetic localization in familiar arenas. (**A**) Uncertainty (blue particle cloud) either oriented or disoriented initially, showing the estimated pose (cyan arrow) was close to the true pose (red arrow) after 90 seconds. For clarity, only the position of each particle's pose is shown. See also Video S1. (**B**) 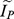 at 48 minutes, 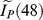, was above chance (0.5) either oriented or disoriented initially (Mann-Whitney U-test, p = 4.3×10^−164^ and p = 7.5×10^−161^, respectively). In both cases, *θ* was non-randomly distributed (Rayleigh test, p<10^−256^) and centred on the true direction (circular m test, p = 0.80 and p = 0.84, respectively) following 48 minutes. 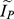 (solid lines) and *V(θ)* (dotted lines) both showed incomplete convergence at 48 minutes (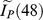, Wilcoxon test, p = 2.6×10^−5^; κ-test, p<10^−16^). *I_p_* kinetics also differed (disoriented, *t_90_* = 2:18; oriented, *t_90_* = 4:32). (**C**) Simulated grid cell spikes during idiothetic localization in 4 arenas with 1-fold rotational symmetry, initially disoriented (rows 2–5), and initially oriented (row 1). Autocorrelograms of the normalized firing fields from 46–48 minutes are included (right column). See also Table S1. (**D**) Median *I_p_* and *V(θ)* functions (left), and *I_p_(48)* distributions (right) are shown for the four arenas in (**C**) when initially disoriented.

Figure 2A shows snapshots of the positional uncertainty distribution along random trajectories, combining idiothetic self-motion cues with arena boundary memory. Starting either oriented or disoriented, the true pose remained close to the estimated pose. 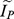 remained above 0.5 using idiothetic cues (Fig. 2B) demonstrating localization success. Similarly, the directional component of the pose estimate, *θ*, was centred on the true direction. Not surprisingly, initial orientation improved position estimation but its effect on *I_p_* was no longer detectable at 96 minutes (Wilcoxon test, p = 0.49). Likewise, *V(θ)* remained consistently higher when initially disoriented (Fig. 1B, dotted lines), but the effect persisted beyond 192 minutes (κ-test, p = 6.0×10^−4^). Lastly, 90% of changes in 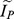 occurred within the first 5 minutes. Together, these results show that idiothetic localization was achieved rapidly even when initially disoriented.

### Sufficient spatial information to express location estimates as grids

To determine whether the idiothetic localization described above could sustain spatially selective firing patterns similar to those of grid cells, a stochastic spiking model of grid cells was used [17] (SI Modelling and Analysis). Accurate localization was expected to result in multiple distinct, spatially regular activity peaks (modes).

Distinct grids were seen both when the animal was initially oriented (Fig. 2C, row 1) and initially disoriented (Fig. 2C, row 2). Autocorrelograms (Fig. 2C, right column) of the normalized firing fields showed spatial regularity similar to grid cells [13], [18], [19]. Of note was the rapid emergence of the grid pattern during the first 2 minutes (Fig. 2C, 0–2) when initially disoriented, consistent with the changes in *I_p_* and *V(θ)* of Fig. 2B. These results show that it is plausible for neural correlates of successful idiothetic localization to be observed using arena size and timescales similar to rodent experiments.

### Arena properties allowing idiothetic localization

A range of boundary properties were found to be compatible with idiothetic localization, including one axis of reflective symmetry (all arenas of Fig. 2C), arena concavity (T-maze arena), lack of vertices and straight edges (egg-shaped arena), or a circular outer arena boundary (void landmark arena). In all cases, the spatial information content and gridness indices (Table S1) demonstrated spatial specificity comparable to published rodent place and grid cell data [19], [20]. However, localization metrics including 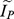 (Fig. 2D, left) and *I_p_* distributions (Fig. 2D, right) varied with arena, showing that idiothetic localization performance depended on arena geometry. Noise level, additional allothetic boundary contact information and arena size also had graded effects on localization performance (Text S1 - Supporting Results, Fig. S1 and S2, Table S1).

**Figure 3:**
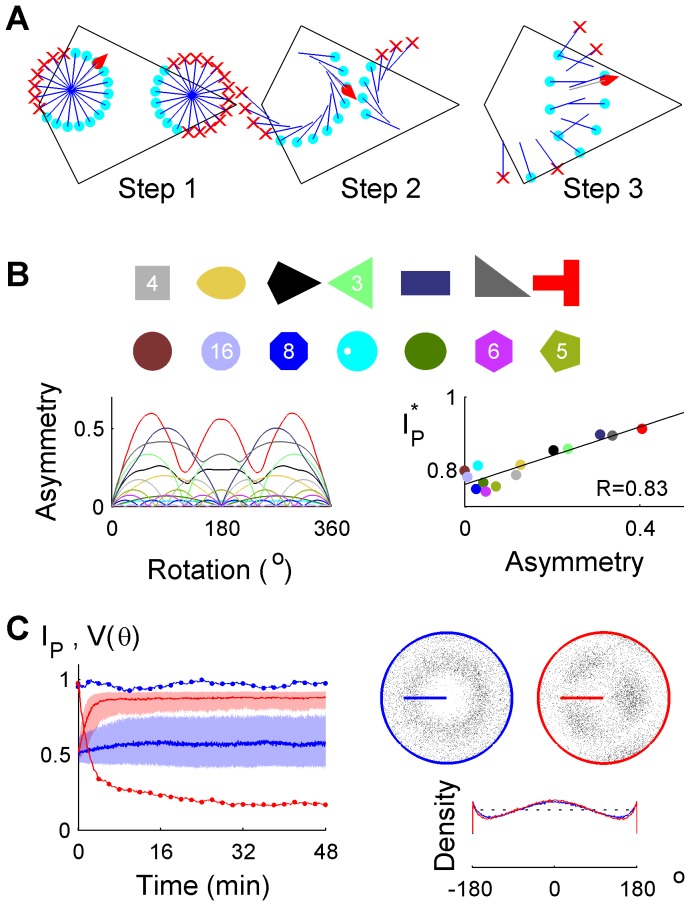
Boundary properties affecting idiothetic localization. (**A**) Culling of pose hypotheses over 3 true steps (red pointer), starting with 20 possible directions and two possible positions (40 poses). Each pose hypothesis independently tracked the displacements (blue) using only idiothetic displacement information. Pose estimates which crossed a boundary (red crosses) were removed. Acceptable hypotheses (cyan dots) were cloned to maintain the total particle population constant. For clarity, no sensory noise was included in this illustration but was present in all simulations. (**B**) Arenas of equal area (top) in ascending order (bottom to top, left to right) of rotational asymmetry functions (lower left) averaged over 360°. The adjusted I_p_^*^ following 48 minutes increased with mean asymmetry (lower right). Regular polygonal arenas are labelled by the number of edges. (**C**) *I_p_* (median, IQR) and *V(θ)* are shown using traversable space (blue) or boundary crossing (red) to update the pose estimate in a circular arena with an asymmetric barrier. Simulated grid cell spikes using a discrete model which did (right) or did not (left) use boundary crossing (46–48 min). There was a uniform angular distribution in traversable space (dotted line) but not traversed positions (solid lines).

The mechanism of localization from initial disorientation may be intuited by considering the mechanism of action of a particle filter. Among a large number of initially random pose hypotheses (represented by particles), some are close to the true pose while others are not (Fig. 3A, left). A poor initial pose estimate is more likely to result in an estimated trajectory which crosses a boundary in memory, compared to a good initial pose estimate (Fig. 3A, middle and right).

Over time, estimates cluster around the true pose, plus any rotationally symmetric poses. The latter occurs because in arenas with *n*-fold rotational symmetry (*n*-RS), there are *n* poses which are geometrically equivalent and consistent with the boundary map, making idiothetic localization to a unique pose impossible. Therefore the combination of boundary asymmetry and an internal model of boundary crossing sufficed for idiothetic localization.

### Predictions of idiothetic localization

Through careful inspection of the particle filter model, five further predictions related to idiothetic localization were made and demonstrated through simulations using the rodent HD error model. Firstly, local arena geometry was expected to affect the *I_p_* value differently in arenas with the same degree of rotational symmetry, since within each of *n* sectors of an arena with *n*-RS, localization should be possible with performance depending on the sector's asymmetry. An adjusted I_p_* for *n*-RS arenas was found to be positively correlated to the average rotational asymmetry (Fig. 3B, Text S1 - Modelling and Analysis), confirming that both local and global rotational asymmetry affected localization performance.

A second prediction was that an asymmetric interior barrier could replace rotational asymmetry in the traversable space, and was tested in a circular arena (Fig. 3C). Modelling boundary crossings, a circular traversable area (*∞*-RS) allowed idiothetic localization when an internal barrier was present. However, localization failed when estimated movements were represented as discrete steps which ignored barrier crossings (Text S1 - Modelling and Analysis), showing that the way that self-motion cues and arena memory information are combined can significantly affect performance. In this instance, being able to determine whether a boundary has been crossed was more important for the navigation system than merely determining whether it remained inside the arena. In the particle filter, the pose hypotheses (particles) which crossed any boundary were removed, even if they remained within the traversable space of the remembered arena. A related prediction was that intermittent use of a compass suffices for localization in empty circular arenas (Fig. S4), since a compass directly breaks rotational symmetry in any arena. In this way, allothetic compass information may be incorporated infrequently, while place information is maintained using idiothetic cues for most of the time. Importantly, no ‘reset’ of the position estimate was required – only breaking of rotational symmetry through the compass.

**Figure 4:**
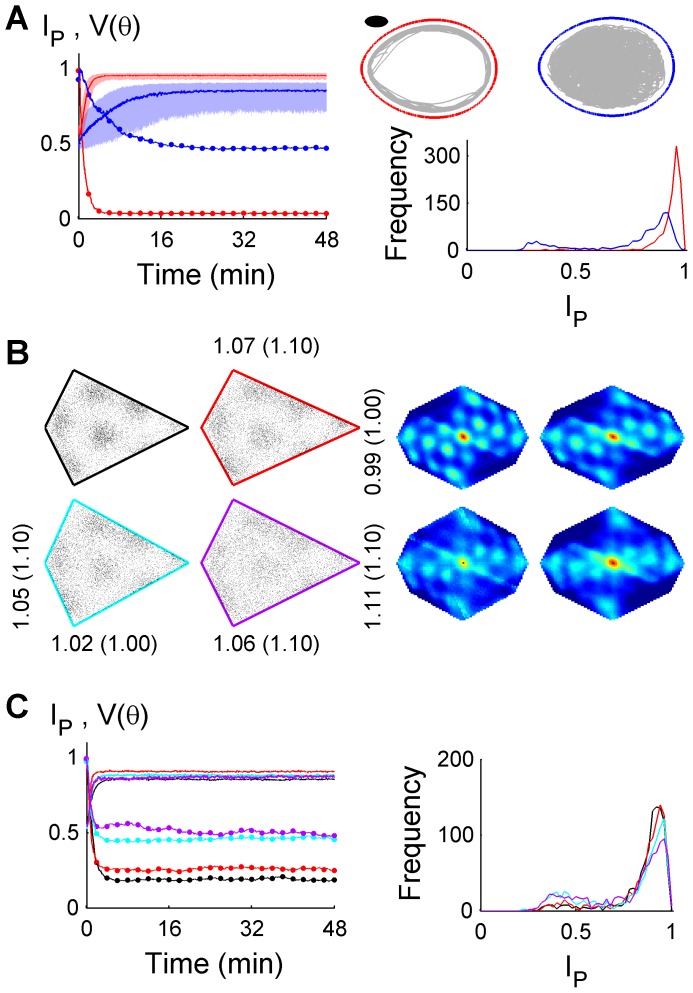
Other properties affecting idiothetic localization. (**A**) *I_p_* (median, IQR) and *V(θ)* during idiothetic localization (left) by a 7×15 cm elliptic navigating agent (black), during random (blue) or thigmotactic (red) movements in an egg arena. The thigmotactic movement strategy resulted a higher 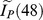 (right, Wilcoxon test, p = 1.3×10^−200^) and lower *V(θ)* (κ-test, p<10^−16^) at 48 minutes, and faster *I_p_* rise kinetics (random t_90_ = 13:25; thigmotactic t_90_ = 2:46). An example trajectory is shown for each movement strategy (grey). See also Video S3. (**B**) Idiothetic localization following linear arena expansion. Simulated grids partially rescaled with the expansion of the test arena, assuming the standard kite boundary in memory. The optimal scaling factors determined by the normalized firing field are shown together with the true arena scale factor (parentheses). See also Table S1. (**C**) Median *I_p_* and *V(θ)* functions (left), and *I_p_(48)* distributions (right) are shown for the four arenas in (**B**). *I_p_* functions were calculated using the test arena.

A third prediction was that the centre of a finite-sized navigating agent need not reach the arena boundary, if it used an internal model of its own perimeter. This was demonstrated using an elliptic agent using both random and thigmotactic (wall following) trajectories (Fig. 4A). Accuracy was significantly improved by using a thigmotactic movement strategy, demonstrating trajectory-dependence of localization performance.

A fourth prediction was that the test arena may vary slightly from the learned arena. Assuming a standard kite arena in memory, the test arena was linearly expanded in the *X*, *Y* or both *X* & *Y* directions, by 10% (Fig. 4B, S3C). Grid modes showed greater spatial specificity following *X* or *Y* expansion, than along both directions. Partial grid rescaling was observed [19], principally along the expansion direction. These results show that strict congruence between the learned and test arena was unnecessary, but that spatial specificity was affected by a disparity between the learned and actual boundary.

**Figure 5:**
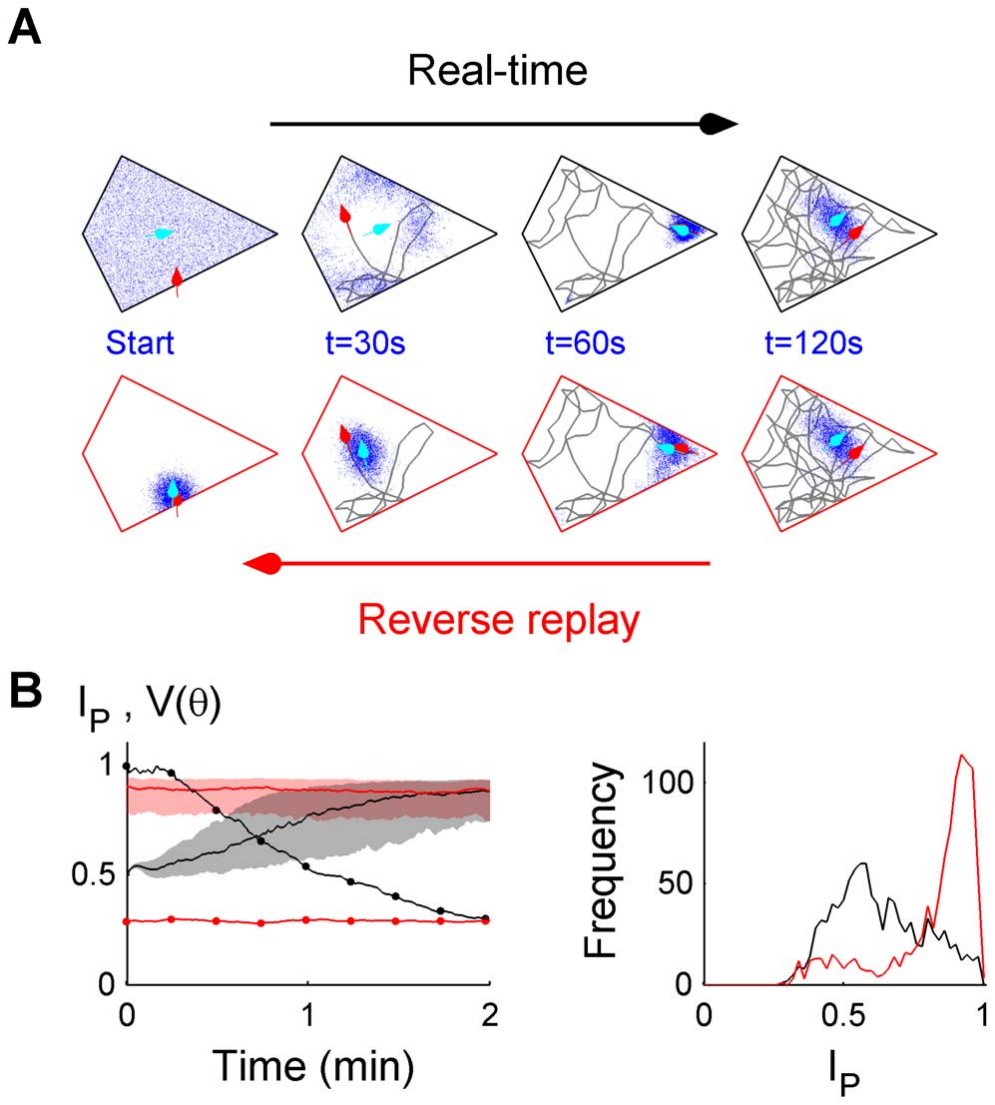
Estimating initial pose using reverse replay of past self-motion estimates. (**A**) Uncertainty (blue particle cloud) during real-time localization or during reverse replay, showing the estimated pose (cyan arrow) and the true pose (red arrow) during a two-minute period initially disoriented. (**B**) *I_p_* (median, IQR) and *V(θ)* are shown during real-time localization (black) or during reverse replay (red), and the corresponding *I_p_* distributions at 30 s.

A final prediction was that the true pose at the beginning of a disoriented trial can be recovered by replaying self-motion estimates in reverse. In real time, the initial pose estimate was uniformly distributed over the arena in all directions. Following a period of localization, the final pose estimate was treated as the initial pose estimate of the same trajectory replayed in reverse, in an ‘offline’ manner. Fig. 5 shows that following reverse replay, pose estimates were substantially improved from real-time pose estimates during initial localization, which were optimal at the time. Assuming that a sequence of self-motion estimates can be stored and retrieved later, this simple strategy can significantly improve a past pose estimate retrospectively. Alternatively, an ‘online’ backward inference procedure can also be used to achieve retrospective localization for a chosen time, without storing self-motion estimates (Fig. S5, Text S1 – Modelling and Analysis, Text S1 - Supporting Results). The ability to accurately recover the starting pose implies that homing is possible using only idiothetic sensory cues, even when initially disoriented. In principle, direct homing can occur after an indefinite period of time, since both current pose and initial pose (‘home’) can be determined.

## Discussion

### Idiothetic localization is a dynamic process

In terms of a particle filter, idiothetic localization can be seen as a consequence of a dynamic competition between increasing uncertainty due to iPI errors (Fig. 1A) and decreasing uncertainty due to culling of invalid hypotheses which cross a boundary (Fig. 3A). The rate of increase of uncertainty (diffusion-like particle cloud expansion) depends on the magnitude of intrinsic iPI errors (Fig. S1) and path structure (e.g., path tortuosity [16], arena size and shape). The rate of decrease of uncertainty (i.e., particle culling) depends on the interaction of arena shape (e.g., Fig. 2), size (e.g., Fig. S3) and path structure (e.g., Fig. 4). When the factors affecting the rate of increase and decrease in uncertainty are kept constant over a prolonged period of time, a dynamic equilibrium is reached which corresponds to the plateau seen in most 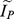 functions.

The complex interactions between arena shape, size and path structure are briefly described. Intuitively, if relatively few pose hypotheses (particles) can account for a sequence of displacements, uncertainty tends to decrease. For example, a path taken from the acute to obtuse to a right angle corner of a kite arena cannot be accounted for by any particle trajectory except one which is close to the true trajectory. In contrast, there are infinite possible paths which span the diameter of a circular arena or follow its boundary, so there are always a range of particle trajectories which can account for any displacement sequence arising in a circular arena. Consequently, determining a unique location is impossible. In a given 1-RS arena, the path structure determines the relative uniqueness of the displacement sequences. Hence very few thigmotactic loops around a 1-RS arena (e.g., Fig. 4A) are required to uniquely determine location, within the limits of noise. Therefore, when rotational asymmetry is due to an outer boundary, thigmotaxis is an efficient strategy for localization. In contrast, a trajectory biased towards the center of such an arena takes longer for localization, and the agent spends relatively longer time periods poorly localized leading to a lower level of equilibrium performance. Thus large arenas tend to increase uncertainty since more time is spent in the centre of the arena. This effect is exacerbated by the nonlinear increase in positional uncertainty due to unconstrained iPI [16]. It is important to note that these effects are not applicable if the rotational asymmetry is due only to an interior arena structure such as a barrier or void. In this case, thigmotaxis is not sufficient for localization, while a trajectory bias towards the arena centre will improve localization compared to a random trajectory.

The magnitudes of the competing rates of increase and decrease in uncertainty also depend on the initial pose distribution (e.g., Fig. 2A, S2). For example, when initially disoriented, a large portion of pose hypotheses are grossly incompatible with self-motion cues given the known arena, resulting in a relatively high rate of decrease in uncertainty (particle culling) relative to the increase from iPI (diffusive expansion of particle cloud). Consequently, overall uncertainty decreases, leading to improved localization when initially disoriented. In contrast, when initially oriented, the majority of pose hypotheses remain compatible with self-motion information so the rate of particle culling is low relative to the diffusive expansion of the particle cloud due to iPI errors. Hence, there is an overall decay in localization performance when initially orientated. In general, the direction of change in localization performance (e.g., 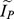) depends on whether the current pose uncertainty is above or below the equilibrium level. In turn, the equilibrium performance level depends on the interaction between iPI errors, arena size, shape and path structure.

### Arena rotational asymmetry and boundary maps

By itself, rotational asymmetry is not sufficient for long term localization. Fig. 1B shows that even combining two levels of rotational symmetry breaking (compass+kite arena) is not sufficient for localization. The lack of an arena map led to unlimited growth of uncertainty in the position estimate, despite accurate orientation. In contrast, breaking rotational symmetry in combination with an arena map allowed long term localization (e.g., Fig. 2, Fig. S4). Hence it is the combination of rotational asymmetry and arena map which allows idiothetic localization.

However, an arena map is not always necessary if allothetic cues are available. When detected, the acute and obtuse corners of a kite arena are unique landmarks which can theoretically be used by a modular navigation system for localization [17], [21], without requiring a full boundary map in memory. The axis of the two unique landmarks provides the symmetry breaking information (hence orientation), while the point-like nature of each landmark provides location information. Similarly, rats and other animals can leave markings inside arenas which can potentially be used to break rotational symmetry and allow localization even in a circular arena, without a boundary map. Hence it is specifically the restriction to using only idiothetic cues which necessitates both the use of an arena map and presence of rotational asymmetry for successful localization.

### Implications for spatial navigation mechanisms

It was shown previously that combining self-motion cues and arena memory significantly slowed the decay of pose estimates in a circular arena, when initially oriented. When allothetic boundary contact cues were included, the residual localization achieved could account for the prolonged stability of rodent place and grid fields observed in darkness despite an unstable head direction system [17]. It is now shown that in arenas with 1-fold rotational symmetry, localization decay can in fact be stopped altogether, that allothetic cues are not necessary, and the navigating agent can be initially disoriented. Important implications of these new findings are discussed below.

Using only idiothetic cues in conjunction with information already in memory potentially reduces computational load during navigation. Such a strategy allows a navigating agent to devote computational or attentional resources for processing allothetic sensory information for other tasks. Although precision is reduced, it is likely to be a low-risk strategy since occasional use of allothetic cues suffices to recover near-optimal localization (e.g., Fig. S2A, Fig. S4). There is evidence that animals may use allothetic sensory cues intermittently during navigation [22], [23]. If allothetic cues are used intermittently during localization by the hippocampal-entorhinal space circuit, two consequences may be observed. Firstly, cumulative spatial uncertainty may increase spatial firing variability beyond that expected from average firing rates. Over multiple passes through the same true location, positional uncertainty will cause variability in the estimated location, potentially reflected in variability in spike activity. This may contribute to the phenomenon of ‘overdispersion’ [24], [25] observed in CA1 place cells, whose firing fields are influenced by both allothetic cues [26], [27], and self-motion information such as via grid cells [8], [28]. A second possible consequence of cumulative spatial uncertainty is temporary divergence in the spatial code relative to allothetic landmarks. A temporary divergence may contribute to the multiple ensemble place codes which have been reported in rodent CA1, interpreted as alternating attention between distal landmark cues and self-motion cues [25]. However, the results of the present study suggest that using self-motion cues alone is likely to lead to degradation of the place code within 2–3 minutes (Fig. 1). Hence in the long term, attention to self-motion cues is not sufficient to account for a second stable place code, unless there is also intermittent compass information, rotational asymmetry in the arena, or both.

The rodent medial entorhinal cortex (mEC) contains both border cells and grid cells [13], [29], which raises the possibility that it could be a self-sufficient localization system. If mEC border cells encode a boundary spatial representation, then together with the putative grid cell based path integration system [8], [13], fusion of self-motion and boundary memory information should enable localization. If so, stable grid fields are expected to emerge in familiar arenas with 1-fold rotational symmetry, in the absence of vision, initially disoriented, and in a hippocampus-independent manner. However, in arenas with >1-fold rotational symmetry such as circular or square arenas, such a system would require supplementation with a compass cue, at least sporadically (e.g., Fig. S4). In rodents, the latter could be provided via the visually-stabilised head direction (HD) system [30]–[32], whose firing properties mature prior to place and grid cells during development [33], [34], as would be expected if HD cells provided important symmetry-breaking information for place and grid cells.

Boundaries occurring in natural environments rarely have >1-fold rotational symmetry, making it plausible that biological navigation systems may exploit this property for localization. It remains to be tested whether species which spend significant time in enclosed spaces [35]–[37] are more likely to have evolved mechanisms to use this localization strategy. It has been reported previously that the persistent stability of rodent place and grid cell firing in darkness starting from full orientation [12], [13] is likely to rely on the fusion of self-motion and boundary information [17]. Assuming the same type of information fusion occurs starting from full disorientation, the results of the current study suggest the emergence of stable place and grid fields should occur in arenas with 1-fold rotational symmetry.

It is worth noting that the localizing mechanism described here need not be restricted to bounded spaces with impassable physical barriers. For instance, this strategy is equally applicable if an animal's trajectory is limited by a few distinct landmarks, forming a virtual arena with 1-fold rotational symmetry in an otherwise open trajectory space. Familiar landmarks could thus be used to break rotational symmetry in the trajectory space without a physical barrier, and resulting in successful localization without knowledge of the distance or allocentric direction to those landmarks during a journey. However, the geometry of the virtual boundary must be known. In this hypothetical example, the trajectory is guided by allothetic cues, while localization uses idiothetic cues and information already in memory.

One prediction of idiothetic localization was that reverse replay of past information enabled retrospective improvement in localization (Fig. 5). That is, knowledge of current location improved when future information became available. Experimentally, replays of sequences of place cell activity corresponding to past behavioural trajectories have been reported during sleep [38], [39] and when awake [40], including in reverse temporal order [40], [41]. The modelling results here suggest that a possible role of hippocampal reverse replay may be to improve past estimates of location, which may in turn improve the accuracy of future path planning [42].

While the present study examined the information and computations which may be necessary and sufficient to be used in conjunction with a known boundary map for localization, future studies will need to address the acquisition of the boundary map itself. In robotics, particle filter methods have been used successfully to build boundary maps using only self-motion and boundary detection cues, starting from full disorientation – a Simultaneous Localization and Mapping (SLAM) problem [43], [44]. Similar methods may be used to investigate the factors which could affect the acquisition of a boundary map under biologically realistic conditions, and make predictions about localization performance when only imperfect maps are available.

### Applications in arena-based experiments

From the findings in this study, it is proposed that spatial memory systems which can effectively combine idiothetic self-motion cues and boundary memory can determine location in familiar arenas with 1-fold rotational symmetry. If allothetic cues are stringently removed, localization necessarily demonstrates the fusion of idiothetic self-motion and memory-based boundary information. This prediction may be tested, for example, by using blindfolded human subjects passively led along random or thigmotactic trajectories. Where *in vivo* recordings are feasible, it may be possible to isolate the cells and circuits where the fusion of idiothetic self-motion and boundary memory information occurs. For instance, if stable mEC grid fields emerge from full disorientation under the cue-restricted conditions described, information fusion must occur either at or upstream of the medial entorhinal grid cells.

Together, the reported results reveal that detection of cues from the external world is not always necessary for localization, that bounded arenas are distinct from true open fields [9], [10], [45], [46], and that any information which breaks rotational asymmetry may be useful for localization. Furthermore, arena boundaries affect navigational difficulty in a size-, shape- and path- dependent manner, and need to be addressed during the design and interpretation of experiments which investigate the navigational abilities of animals in arenas. Finally, the results suggest that specific arena designs can be used to interrogate the combination of self-motion and memory information in the hippocampal-entorhinal space circuit, whose properties are influenced by environmental boundary information [18], [29], [47], [48].

## Methods

It is known from recordings of Head Direction (HD) cells in rats, that error in the estimate of head direction increase steadily as the animal moves away from the place where it was first deprived of its vision [30], [31]. The accumulation of errors in head direction was modelled as a Wiener process [17], based on the reported drift in tuning functions of HD neurons without vision [30], [31]. Simulated rodents began each trial either oriented (i.e. with perfect initial pose information) or disoriented (no initial pose information).

A particle filter was used to approximate Bayes-optimal fusion of idiothetic self-motion and boundary information, to provide the best estimate of successive poses given noisy displacement inputs. The localisation performance, as revealed by this particle filter approach, was analysed across 10^3^ random trials in each condition, using metrics developed previously to characterise instantaneous spatial accuracy and precision. Briefly, the median place stability index, 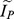, characterizes the particle filter's localization performance across trials, where 0.5 represents chance (uniform uncertainty within the arena), and 1 represents perfect localization. Similarly, the circular variance 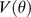 measures the particle filter's directional performance across trials, where 0 represents uniformly random heading estimates, and 1 represents no heading error.

The particle filter estimate of position was then used to simulate the firing activity of grid cells in medial entorhinal cortex, using a stochastic spiking model where spike probability decreased with estimated distance from the neurons' preferred firing locations [17]. The resultant spike patterns were analysed using standard time-averaged metrics developed to characterize place and grid cell activity [19], [20], including information content and gridness indices. See Text S1 - Modelling and Analysis for further details.

## Supporting Information

Figure S1
**Matched and mismatched uncertainty.** Effects of matched and mismatched angular and linear uncertainty on idiothetic localization. 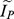 and *V(θ)* functions (left), *I_p_(48)* distribution (middle), simulated grid cell spikes (top right) and firing field autocorrelograms (bottom right) during 46–48 minute period in kite-shaped arenas. **A**, Matched angular uncertainty using 0.25× (light blue), 0.5× (dark blue), 1× (black), 2× (dark red), and 4× (light red) the standard 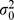. **B**, Matched linear uncertainty using 0.25× (light blue), 0.5× (dark blue), 1× (black), 2× (dark red), and 4× (light red) the standard 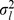. **C**, Mismatched angular uncertainty using 0.25× (light green), 0.5× (dark green), 1× (black), 2× (dark purple), and 4× (light purple) the standard 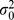. **D**, Mismatched linear uncertainty using 0.25× (light green), 0.5× (dark green), 1× (black), 2× (dark purple), and 4× (light purple) the standard 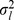. **E**, Boxplots of *I_p_(48)* showing the effects of matched and mismatched angular and linear uncertainty on idiothetic localization performance (outliers not shown). Red bars indicate comparisons of 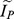 using Wilcoxon test with Holm-Šidák correction (* = p<0.05, ns = not significant). Black bars indicate comparisons of circular concentration of 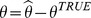 (error in pose direction estimate) following 48 minutes, using κ-test with Holm-Šidák correction (* = p<0.05, ns = not significant).(TIF)

Figure S2
**Intermittent boundary contact.** **A**, *I_p_* (median and IQR) and *V(θ)* functions without vision in a kite-shaped arena, switching from idiothetic cues only (orange bars), to idiothetic cues plus boundary contact (yellow bars) in 8 minute blocks, initially oriented (green) and disoriented (black). Boxplots compare the residual effects of orientation versus disorientation, and intermittent (+) versus no (−) wall contact information on 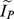 (Wilcoxon test with Holm-Šidák correction, * = p<0.05, ns = not significant). The data using no wall contact were from Fig. 2B. 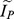 was higher for all conditions with initial orientation (green) relative to initial disorientation (black) showing that initial pose information had a robust and significant residual effect on localization. In contrast, 16 minutes of wall contact information (40+) had no residual effect on 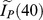 compared to no wall contact information (40-). **B**, Simulated grid cell spikes and firing field autocorrelograms from **A**, initially oriented (top row) and initially disoriented (bottom row), with (right) and without (left) boundary contact. Consistent with the 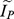 results of **A**, grids showed higher spatial specificity using boundary contacts while initial orientation showed residual effects beyond 30 minutes.(TIF)

Figure S3
**Large or discrepant arenas.** **A**, 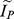 and *V(θ)* functions (left) and *I_p_(48)* distribution (right) using a kite arena 4-fold in area (red) compared to a standard kite arena (black). **B**, Quadrupling the area of the kite arena resulted in no distinguishable grid modes at 46–48 minutes (first column) when the standard 30 cm grid spacing was used in spike simulation. Doubling the grid spacing (linear scaling, second column) recovered grid modes, which were less distinct than in the standard kite arena (third column). The crosscorrelogram (fourth column) between the normalized firing field of the standard and the 4×-arena (double grid spacing) yielded a gridness index of 0.39. Scale = 50 cm.(TIF)

Figure S4
**Idiothetic localization combined with an intermittent noisy compass in circular arenas.** **A**, *I_p_* (median and IQR) and *V(θ)* using a compass stochastically, averaging once every 30 s with Gaussian measurement error 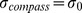, in a 76 cm (black) and 152 cm (blue) diameter circular arena. *I_p_(48)* distributions were similar (right), as were kinetic parameters (76 cm, *t_90_* = 10.1 s; 152 cm, *t_90_* = 12.4 s). Although absolute differences were small, median *I_p_* was significantly higher (Wilcoxon test, p = 1.2×10^−10^) and *V(θ)* was significantly lower (κ-test, p = 8.1×10^−6^) following 48 minutes in the 152 cm arena. **B**, Simulated grid spikes (top row) and autocorrelograms (bottom row) using 30 cm grids (left and middle), and 60 cm grids (right), in 76 cm (left) and 152 cm (middle and right) diameter circular arenas.(TIF)

Figure S5 **Improved retrospective localization using either offline reverse replay (beta recursions) or online backward inference (gamma recursions).** (**A**) Uncertainty (blue particle cloud) during real-time localization (row 1) and during offline reverse replay (row 2), showing the estimated pose (cyan arrow) and the true pose (red arrow) during a two-minute period initially disoriented. Row 3 shows the same path, using online backward inference to estimate the initial pose (time *0*). The particle size shown is proportional to the number of parent particles with identical poses. (**B**) *I_p_* (median, IQR) and *V(θ)* are shown during real-time localization (black), during offline reverse replay (red), and during online backward inference (green). The latter shows the online update in estimate of the initial pose. The right panel shows the corresponding *I_p_* distributions at time *0* following the completion of the two retrospective localization strategies.
(TIF)

Table S1 **Spatial firing properties of simulated grid cells.** Information content (bits/spike) of the top three grid modes by spike count, and gridness indices of simulated grids.
(DOCX)

Text S1 **Modelling and Analysis, Supporting Results, and Supporting References.**
(PDF)

Video S1 **Idiothetic localization in a kite-shaped arena during 0 to 8 minutes, initially oriented.** The top panel shows the uncertainty (blue particle cloud) during a random trajectory, comparing the true pose (red dot) and estimated pose (cyan dot) in 3D pose space (X position, Y position, and direction θ). The vertical axis (θ) ranges from 0 to 360°. The lower left panel shows the positional distribution (blue particle cloud), with direction indicated by the red (true) and cyan (estimated) pointers. The lower right panel shows the direction distribution of the particle cloud (blue polar histogram), overlaid on the true direction (red pointer).
(MP4)

Video S2 **Idiothetic localization in a kite-shaped arena during 0 to 8 minutes, initially disoriented.** Otherwise as per Video S1.
(MP4)

Video S3 **Idiothetic localization in an egg-shaped arena during 0 to 8 minutes, initially disoriented, with an elliptic body perimeter.** The top panel shows the uncertainty (blue particle cloud) during a thigmotactic trajectory, comparing the true pose centre (red dot) and estimated pose centre (cyan dot) in 3D pose space (X position, Y position, and direction θ). The vertical axis (θ) ranges from 0 to 360°. The lower left panel shows the positional distribution (blue particle cloud), with direction indicated by the red (true) and cyan (estimated) ellipses. The lower right panel shows the direction distribution of the particle cloud (blue polar histogram), overlaid on the true direction (red ellipse with white arrowhead).
(MP4)
