# Supplementary figures and images for: Estimating Location without External Cues
Source: PLoS Comput Biol. 2014 Oct 30;10(10):e1003927. doi: 10.1371/journal.pcbi.1003927 (PMC4214594; doi:10.1371/journal.pcbi.1003927)

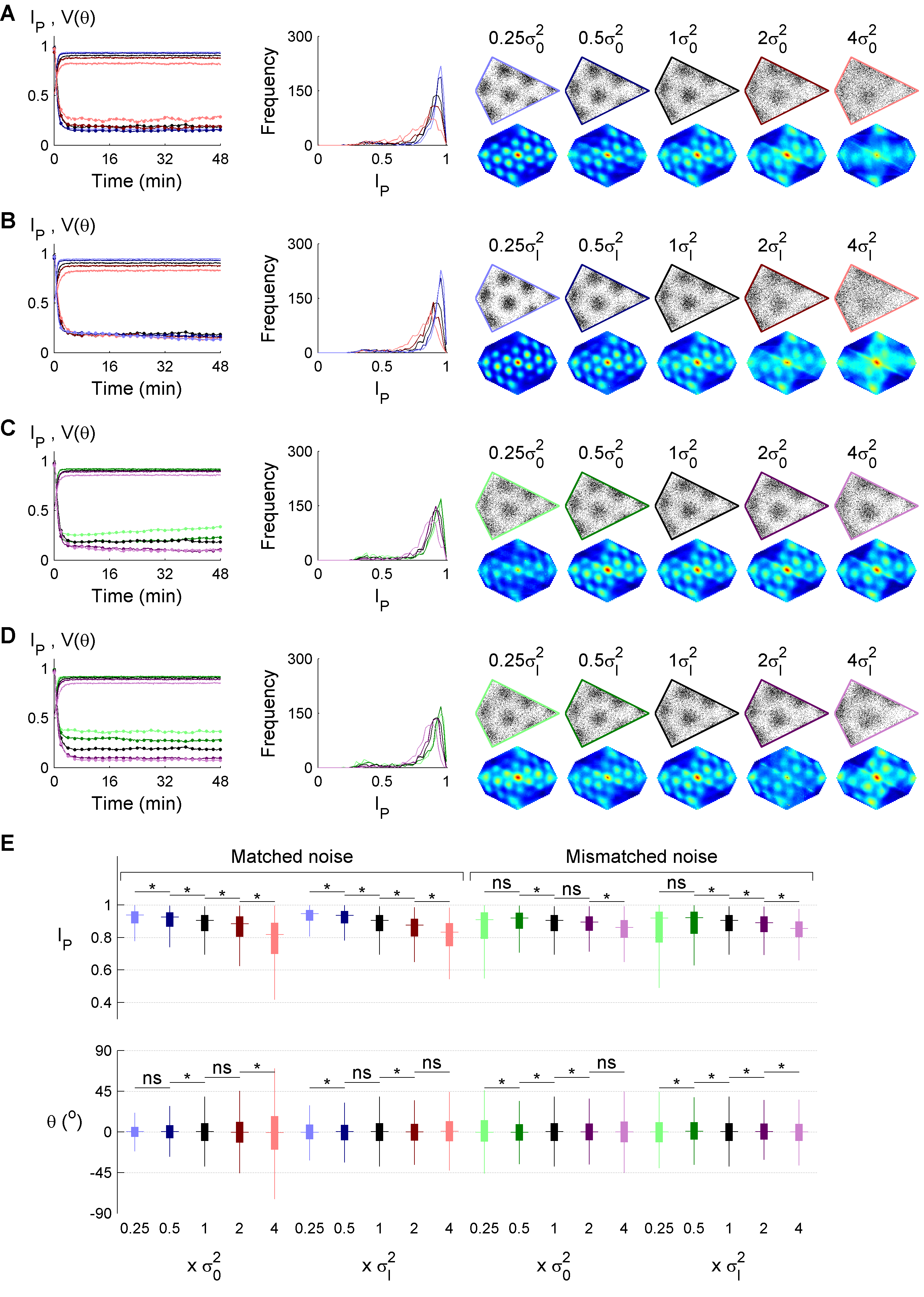

Supplement: Figure S1 — Matched and mismatched uncertainty. Effects of matched and mismatched angular and linear uncertainty on idiothetic localization. and V(θ) functions (left), Ip(48) distribution (middle), simulated grid cell spikes (top right) and firing field autocorrelograms (bottom right) during 46–48 minute period in kite-shaped arenas. A, Matched angular uncertainty using 0.25× (light blue), 0.5× (dark blue), 1× (black), 2× (dark red), and 4× (light red) the standard . B, Matched linear uncertainty using 0.25× (light blue), 0.5× (dark blue), 1× (black), 2× (dark red), and 4× (light red) the standard . C, Mismatched angular uncertainty using 0.25× (light green), 0.5× (dark green), 1× (black), 2× (dark purple), and 4× (light purple) the standard . D, Mismatched linear uncertainty using 0.25× (light green), 0.5× (dark green), 1× (black), 2× (dark purple), and 4× (light purple) the standard . E, Boxplots of Ip(48) showing the effects of matched and mismatched angular and linear uncertainty on idiothetic localization performance (outliers not shown). Red bars indicate comparisons of using Wilcoxon test with Holm-Šidák correction (* = p<0.05, ns = not significant). Black bars indicate comparisons of circular concentration of (error in pose direction estimate) following 48 minutes, using κ-test with Holm-Šidák correction (* = p<0.05, ns = not significant). (TIF) [file pcbi.1003927.s001.tif]

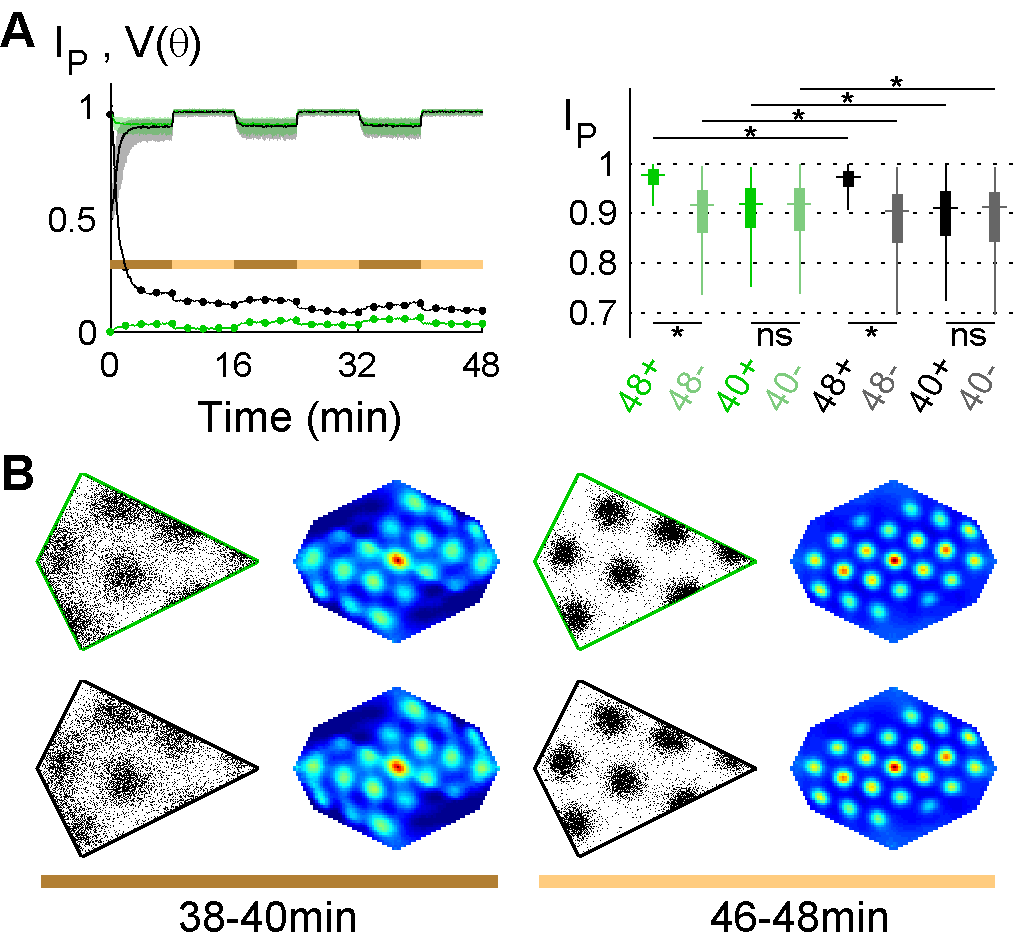

Supplement: Figure S2 — Intermittent boundary contact. A, Ip (median and IQR) and V(θ) functions without vision in a kite-shaped arena, switching from idiothetic cues only (orange bars), to idiothetic cues plus boundary contact (yellow bars) in 8 minute blocks, initially oriented (green) and disoriented (black). Boxplots compare the residual effects of orientation versus disorientation, and intermittent (+) versus no (−) wall contact information on (Wilcoxon test with Holm-Šidák correction, * = p<0.05, ns = not significant). The data using no wall contact were from Fig. 2B. was higher for all conditions with initial orientation (green) relative to initial disorientation (black) showing that initial pose information had a robust and significant residual effect on localization. In contrast, 16 minutes of wall contact information (40+) had no residual effect on compared to no wall contact information (40-). B, Simulated grid cell spikes and firing field autocorrelograms from A, initially oriented (top row) and initially disoriented (bottom row), with (right) and without (left) boundary contact. Consistent with the results of A, grids showed higher spatial specificity using boundary contacts while initial orientation showed residual effects beyond 30 minutes. (TIF) [file pcbi.1003927.s002.tif]

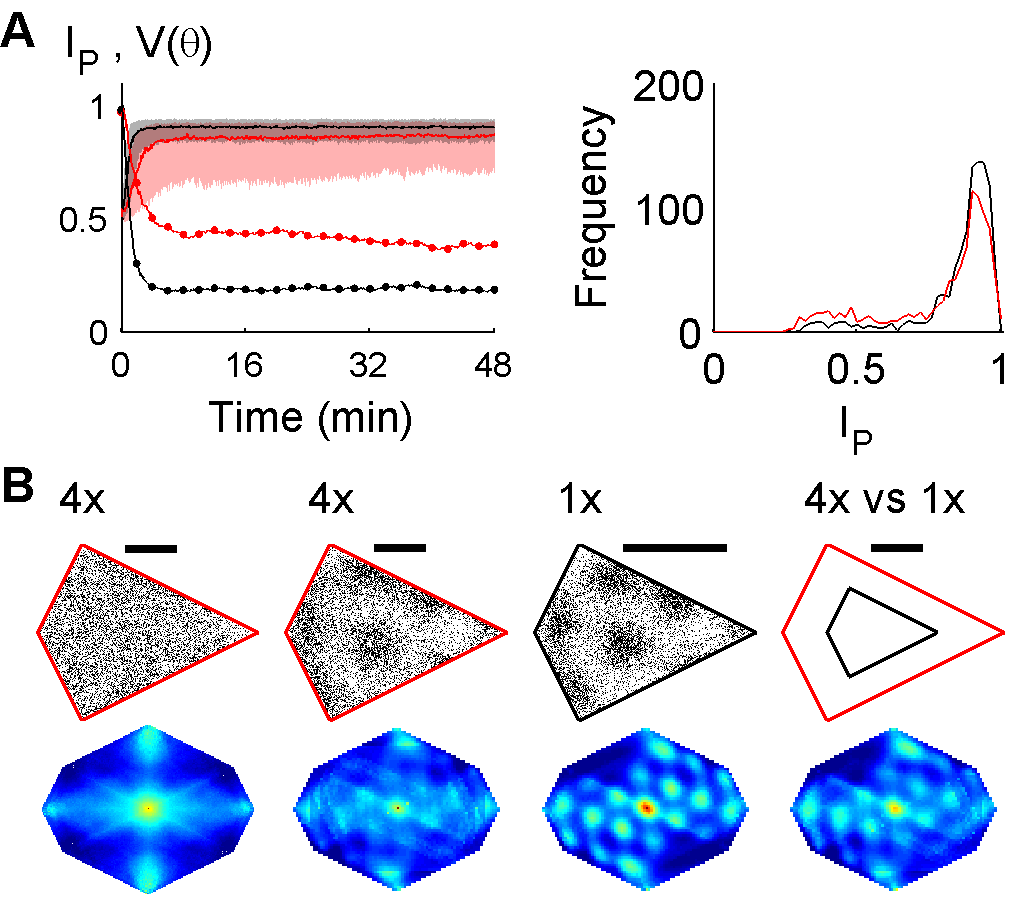

Supplement: Figure S3 — Large or discrepant arenas. A, and V(θ) functions (left) and Ip(48) distribution (right) using a kite arena 4-fold in area (red) compared to a standard kite arena (black). B, Quadrupling the area of the kite arena resulted in no distinguishable grid modes at 46–48 minutes (first column) when the standard 30 cm grid spacing was used in spike simulation. Doubling the grid spacing (linear scaling, second column) recovered grid modes, which were less distinct than in the standard kite arena (third column). The crosscorrelogram (fourth column) between the normalized firing field of the standard and the 4×-arena (double grid spacing) yielded a gridness index of 0.39. Scale = 50 cm. (TIF) [file pcbi.1003927.s003.tif]

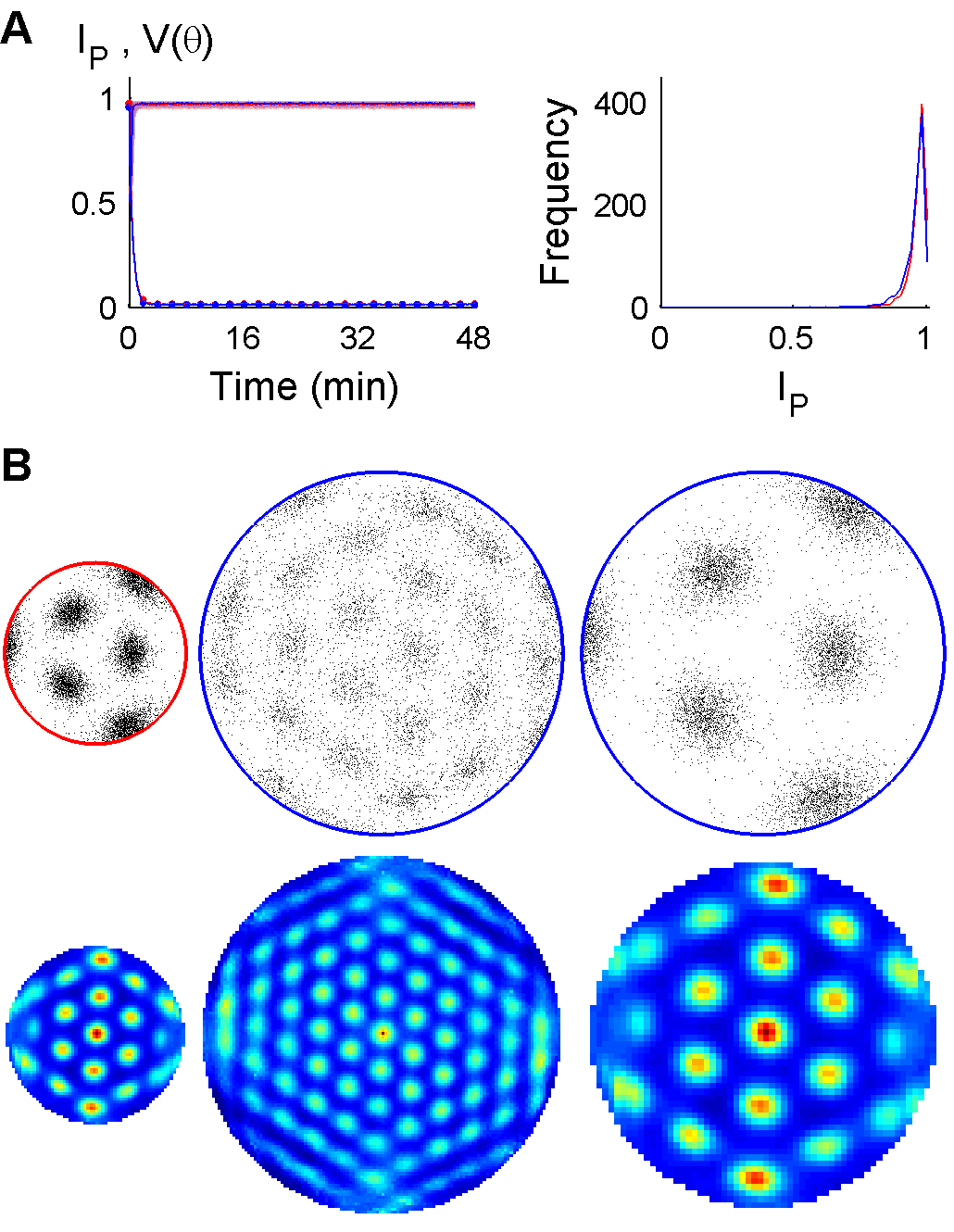

Supplement: Figure S4 — Idiothetic localization combined with an intermittent noisy compass in circular arenas. A, Ip (median and IQR) and V(θ) using a compass stochastically, averaging once every 30 s with Gaussian measurement error , in a 76 cm (black) and 152 cm (blue) diameter circular arena. Ip(48) distributions were similar (right), as were kinetic parameters (76 cm, t90 = 10.1 s; 152 cm, t90 = 12.4 s). Although absolute differences were small, median Ip was significantly higher (Wilcoxon test, p = 1.2×10^−10^) and V(θ) was significantly lower (κ-test, p = 8.1×10^−6^) following 48 minutes in the 152 cm arena. B, Simulated grid spikes (top row) and autocorrelograms (bottom row) using 30 cm grids (left and middle), and 60 cm grids (right), in 76 cm (left) and 152 cm (middle and right) diameter circular arenas. (TIF) [file pcbi.1003927.s004.tif]

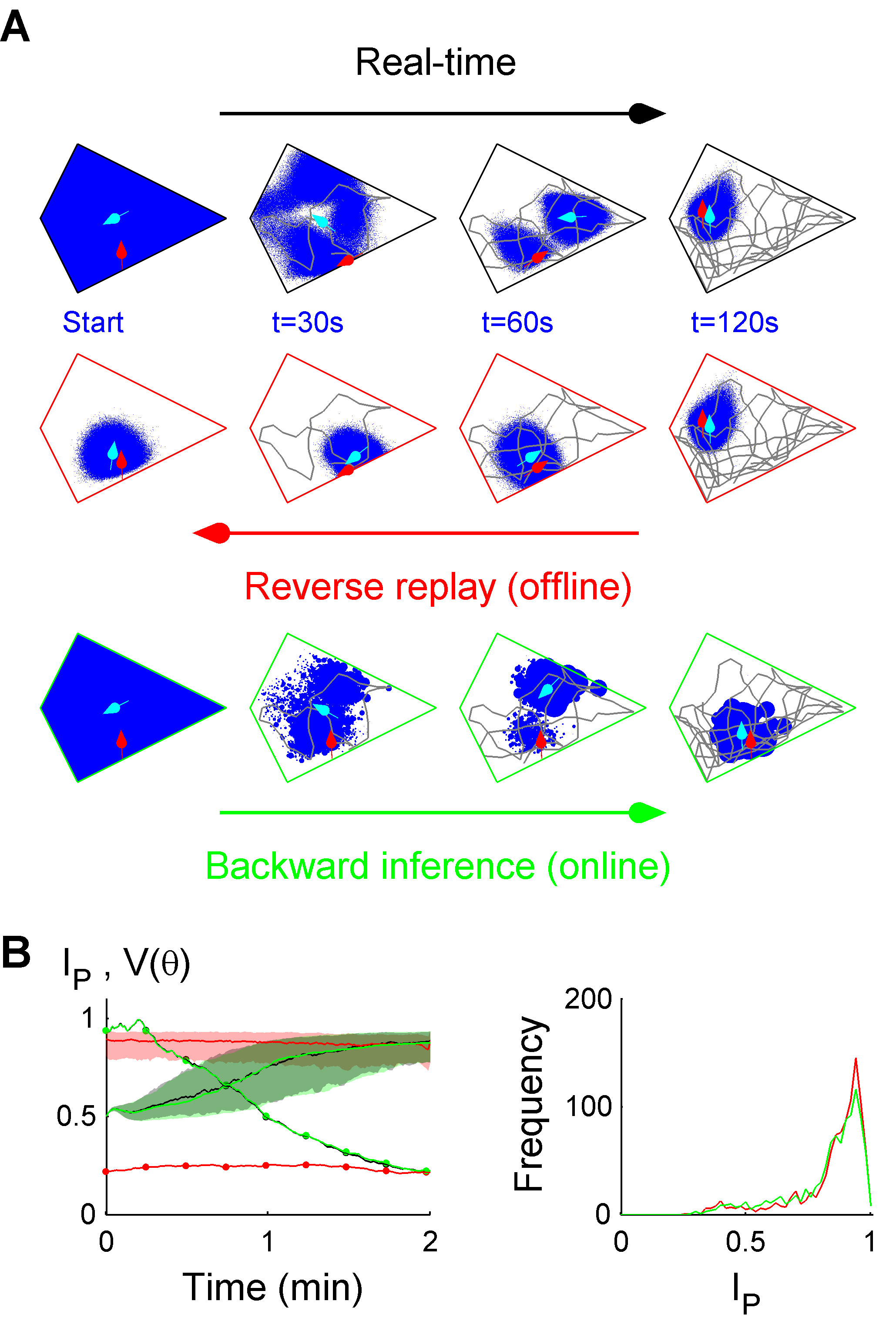

Supplement: Figure S5 — Improved retrospective localization using either offline reverse replay (beta recursions) or online backward inference (gamma recursions). (A) Uncertainty (blue particle cloud) during real-time localization (row 1) and during offline reverse replay (row 2), showing the estimated pose (cyan arrow) and the true pose (red arrow) during a two-minute period initially disoriented. Row 3 shows the same path, using online backward inference to estimate the initial pose (time 0). The particle size shown is proportional to the number of parent particles with identical poses. (B) Ip (median, IQR) and V(θ) are shown during real-time localization (black), during offline reverse replay (red), and during online backward inference (green). The latter shows the online update in estimate of the initial pose. The right panel shows the corresponding Ip distributions at time 0 following the completion of the two retrospective localization strategies. (TIF) [file pcbi.1003927.s005.tif]
