# Supplementary material for: Estimating Location without External Cues
Source: PLoS Comput Biol. 2014 Oct 30;10(10):e1003927. doi: 10.1371/journal.pcbi.1003927 (PMC4214594; doi:10.1371/journal.pcbi.1003927)
Supplement: Table S1 — Spatial firing properties of simulated grid cells. Information content (bits/spike) of the top three grid modes by spike count, and gridness indices of simulated grids. (DOCX) [file pcbi.1003927.s006.docx]

**Table S1**. **Spatial firing properties of simulated grid cells.** Information content (bits/spike) of the top three grid modes by spike count, and gridness indices of simulated grids.

| Figure, condition | Spatial information content | | | Directional information content | | | Gridness index |
| --- | --- | --- | --- | --- | --- | --- | --- |
| 2C, Kite (initially oriented) | 2.2 | 2.2 | 2.5 | 0.06 | 0.03 | 0.04 | 0.63 |
| 2C, Kite (initially disoriented) | 2.1 | 2.0 | 2.2 | 0.03 | 0.05 | 0.05 | 0.75 |
| 2C, T maze | 2.2 | 2.4 | 2.8 | 0.03 | 0.05 | 0.05 | 0.87 |
| 2C, Egg | 2.4 | 2.1 | 2.0 | 0.04 | 0.04 | 0.04 | 1.18 |
| 2C, void LM | 1.5 | 1.7 | 1.8 | 0.02 | 0.02 | 0.03 | 0.63 |
| 3C, no crossing | 0.88 | 0.75 | 0.77 | 0.02 | 0.03 | 0.03 | -0.31 |
| 3C, crossing | 1.9 | 2.0 | 1.9 | 0.03 | 0.05 | 0.03 | 0.83 |
| 3E, standard kite | 2.1 | 2.0 | 2.2 | 0.03 | 0.05 | 0.05 | 0.75 |
| 3E, 1.1X | 1.9 | 1.9 | 2.1 | 0.04 | 0.04 | 0.03 | 0.16 |
| 3E, 1.1Y | 1.4 | 2.0 | 1.7 | 0.04 | 0.03 | 0.06 | 0.38 |
| 3E, 1.1X & 1.1Y | 1.3 | 1.4 | 1.6 | 0.02 | 0.05 | 0.02 | -0.34 |
| S1A, $0.25\sigma_{0}^{2}$ | 2.5 | 2.4 | 2.8 | 0.05 | 0.03 | 0.13 | 1.2 |
| S1A, $0.5\sigma_{0}^{2}$ | 2.4 | 2.3 | 2.4 | 0.02 | 0.04 | 0.03 | 0.88 |
| S1A, $1\sigma_{0}^{2}$ | 2.1 | 2.0 | 2.2 | 0.03 | 0.05 | 0.05 | 0.75 |
| S1A, $2\sigma_{0}^{2}$ | 1.8 | 1.8 | 1.9 | 0.03 | 0.08 | 0.07 | 0.40 |
| S1A, $4\sigma_{0}^{2}$ | 1.3 | 1.5 | 1.7 | 0.02 | 0.08 | 0.10 | -0.40 |
| S1B, $0.25\sigma_{l}^{2}$ | 2.9 | 2.7 | 3.0 | 0.03 | 0.05 | 0.10 | 1.1 |
| S1B, $0.5\sigma_{l}^{2}$ | 2.5 | 2.4 | 2.4 | 0.03 | 0.05 | 0.03 | 1.0 |
| S1B, $1\sigma_{l}^{2}$ | 2.1 | 2.0 | 2.2 | 0.03 | 0.05 | 0.05 | 0.75 |
| S1B, $2\sigma_{l}^{2}$ | 1.9 | 1.9 | 2.0 | 0.04 | 0.04 | 0.07 | 0.47 |
| S1B, $4\sigma_{l}^{2}$ | 1.3 | 1.7 | 1.8 | 0.02 | 0.10 | 0.14 | 0.18 |
| S1C, $0.25\sigma_{0}^{2}$ | 2.0 | 1.7 | 2.0 | 0.03 | 0.04 | 0.07 | 0.25 |
| S1C, $0.5\sigma_{0}^{2}$ | 1.9 | 2.1 | 2.2 | 0.04 | 0.03 | 0.05 | 0.75 |
| S1C, $1\sigma_{0}^{2}$ | 2.1 | 2.0 | 2.2 | 0.03 | 0.05 | 0.05 | 0.75 |
| S1C, $2\sigma_{0}^{2}$ | 2.1 | 2.2 | 2.3 | 0.03 | 0.07 | 0.06 | 0.77 |
| S1C, $4\sigma_{0}^{2}$ | 1.8 | 2.2 | 2.3 | 0.03 | 0.11 | 0.09 | 0.55 |
| S1D, $0.25\sigma_{l}^{2}$ | 2.1 | 1.7 | 1.9 | 0.02 | 0.03 | 0.05 | 0.32 |
| S1D, $0.5\sigma_{l}^{2}$ | 2.2 | 1.9 | 2.1 | 0.03 | 0.04 | 0.10 | 0.47 |
| S1D, $1\sigma_{l}^{2}$ | 2.1 | 2.0 | 2.2 | 0.03 | 0.05 | 0.05 | 0.75 |
| S1D, $2\sigma_{l}^{2}$ | 2.3 | 2.1 | 2.4 | 0.06 | 0.02 | 0.07 | 0.96 |
| S1D, $4\sigma_{l}^{2}$ | 1.8 | 2.2 | 2.4 | 0.02 | 0.13 | 0.19 | 0.91 |
| S2B, oriented (38-40min) | 2.6 | 2.3 | 2.5 | 0.07 | 0.03 | 0.05 | 1.0 |
| S2B, oriented (46-48min) | 4.0 | 3.7 | 3.7 | 0.04 | 0.03 | 0.03 | 1.3 |
| S2B, disoriented (38-40min) | 2.1 | 2.2 | 2.4 | 0.02 | 0.06 | 0.04 | 0.84 |
| S2B, disoriented (46-48min) | 3.5 | 3.3 | 3.5 | 0.02 | 0.02 | 0.03 | 1.3 |
| S3B, large kite (60cm grid spacing) | 1.7 | 1.4 | 1.7 | 0.02 | 0.03 | 0.04 | -0.02 |
| S4B, 76cm | 3.8 | 3.9 | 4.0 | 0.03 | 0.02 | 0.03 | 1.3 |
| S4B, 152cm (30cm grid spacing)^^[[1]](#footnote-1)^#^ | 4.9 | 4.7 | 5.0 | 0.11 | 0.08 | 0.14 | 1.4 |
| S4B, 152cm (60cm grid spacing) | 4.1 | 4.3 | 4.1 | 0.02 | 0.03 | 0.02 | 1.3 |

1. # Spatial information content should be interpreted with care due to a low spike count (<500 for each mode). [↑](#footnote-ref-1)
