# Supplementary material for: Estimating Location without External Cues
Source: PLoS Comput Biol. 2014 Oct 30;10(10):e1003927. doi: 10.1371/journal.pcbi.1003927 (PMC4214594; doi:10.1371/journal.pcbi.1003927)
Supplement: Text S1 — Modelling and Analysis, Supporting Results, and Supporting References. (PDF) [file pcbi.1003927.s007.pdf]

Text S1

Estimating location without external cues

Author: A. Cheung[1*]

Affiliation:

[1]The University of Queensland, Queensland Brain Institute

*Correspondence to: a.cheung@uq.edu.au

Text S1:

Modelling and Analysis

Supporting Results

Supporting References

Modelling and Analysis

Modelling and analysis summary

Recordings of Head Direction (HD) cells in the brains of blindfolded rats clearly show that head direction estimation errors increases steadily as the animal moves away from whence it was first deprived of vision [30,31] . This accumulation of errors in head direction was modelled as a Wiener process [17] . Simulated rodents began each trial either oriented (perfect initial pose information) or disoriented (no initial pose information). A particle filter was used to approximate Bayes-optimal fusion of idiothetic self-motion and boundary information, providing the best estimate of successive poses given noisy displacement inputs. Localization performance was then analysed across 10^3 random trials in each condition, using metrics developed previously to characterise instantaneous spatial accuracy and precision. The particle pose estimate was then used to simulate the firing activity of grid cells, using a stochastic spiking model where spike probability decreased with estimated distance from the neurons' preferred firing locations [17] . The resultant spike patterns were analysed using standard time-averaged metrics developed to characterize place and grid cells [19,20] , summarized in Table S1. An outline of the models and analyses used in this work is shown schematically below. Details are provided in the subsequent sections of the Text S1, as well as the main text.

True trajectory model (random or thigmotactic path)

↓

Rodent error model

↓

Displacement estimates

↓

Particle filter ← Initial information (boundary map, ±initial pose)

← Other information in selected simulations (boundary contact, intermittent compass)

↓ → Videos and video snapshots of particle cloud distribution (representing uncertainty)

Pose estimate → Performance analysis (e.g., place stability index, angular error variance)

↓

Spike model

↓

Grid cell spike pattern → Performance analysis (e.g., gridness index, information content)

Initial information assumptions

Unless otherwise specified, there was no initial pose information (disoriented), and geometrically exact arena boundaries were assumed to be in memory. When initially disoriented, the prior pose estimate was assumed to be a uniform distribution over the arena, and in all directions. The conditions of perfect initial pose information (oriented, e.g., see Fig. 2A, S2A) assumed both true and estimated pose to be exactly at the arena centroid and oriented along the X-axis (0 radians) at the beginning of each trial. During kite arena deformation tests (Fig. 4B), only the standard training arena was assumed to be available in memory.

Random and thigmotactic trajectory model

Pseudocode used to generate true displacement $(\Delta\theta_t, \Delta r_t)$, starting at pose (x_t, y_t, θ_t), for random and thigmotactic trajectory at time step *t* is shown below.

Pseudocode begins

Randomly draw angular displacement $\Delta\theta_t \sim \mathrm{WN}(0, \sigma_\tau^2)$

KeepSearching = true

k=0

While KeepSearching = true **do**

> Randomly draw linear displacement $\Delta r_t \sim \mathrm{N}(\mu_r, \sigma_r^2)$
>
> **If** displacement $(\Delta\theta_t, \Delta r_t)$ from current pose (x_t, y_t, θ_t) requires crossing a boundary
>
> > k=k+1
> >
> > **If** using random trajectory
> >
> > > **If** RandomNumber[#] > 0.1
> > >
> > > > $\tau \sim \mathrm{WN}(0, \sigma_\tau^2)$
> > > >
> > > > $\Delta\theta_t = \Delta\theta_t + \tau(1.1)^k$
> > >
> > > **Else**
> > >
> > > > Find $\Delta\theta_t$ required to head towards centre of arena
> > > >
> > > > $\tau \sim \mathrm{WN}(0, \sigma_\tau^2)$
> > > >
> > > > $\Delta\theta_t = \Delta\theta_t + \tau$

[#] The random value threshold meant that 10% of boundary collisions resulted in turning towards the centre of the arena, while 90% of the time the trajectory was thigmotactic (wall following). This threshold was chosen empirically so that the random trajectory covered the entire arena more or less homogeneously.

End

Else if using thigmotaxis

$$\tau \sim \text{WN}\left(0, \sigma_\tau^2\right)$$

$$\Delta\theta_t = \Delta\theta_t + \tau$$

End

Else

KeepSearching = false

End

End

$$\theta_{t+1} = \theta_t + \Delta\theta_t$$

$$x_{t+1} = x_t + \Delta r_t \cos\left(\theta_{t+1}\right)$$

$$y_{t+1} = y_t + \Delta r_t \sin\left(\theta_{t+1}\right)$$

Pseudocode ends

A new displacement $(\Delta\theta_{t+1}, \Delta r_{t+1})$ can then be generated in the same way starting from the new pose $(x_{t+1}, y_{t+1}, \theta_{t+1})$.

For a finite-sized navigating agent, boundary crossing was deemed to have occurred if any part of the rat perimeter crossed a boundary. For the random trajectory model, $\sigma_\tau = 0.5$, while for the thigmotactic trajectory model, $\sigma_\tau = 0.1$. The factor $(1.1)^k$ ensured that attempted turns gradually increased in magnitude if a viable next step had not been found. WN denotes a wrapped normal distribution. The thigmotactic trajectory model is suitable for any convex arena, but will deviate from concave boundary segments, and will not in general follow interior boundaries or barriers so cannot be considered a general movement strategy useful for idiothetic localization. It should also be noted that despite this algorithm being successful at generating thigmotactic trajectories, there was no boundary contact information *per se* available to the simulated navigation system.

In the random trajectory algorithm, there was a 10% probability of turning towards the arena centre when a boundary was reached, in order to traverse the entire arena. However, it should be noted that boundary contact or centre direction information was not available to the navigation system (particle filter) during idiothetic localization. At any time step *t*, the particle filter was only supplied with an egocentric noisy displacement estimate $\left(\widehat{\Delta\theta_t}, \widehat{\Delta r_t}\right)$, based on the true displacement $(\Delta\theta_t, \Delta r_t)$ obtained from the trajectory algorithm. Therefore with respect to the computations used for idiothetic localization, there was no distinction between using an active trajectory model as shown, versus being passively led along an equivalent path.

It should be noted that the thigmotactic trajectory algorithm was completely agnostic of whether the navigating agent was near the boundary or not. Therefore, the navigating agent need not directly sense the physical boundary to generate a trajectory which then allows successful idiothetic localization. Since the shape and size of the physical boundary affects the resulting trajectory, self-motion cues are necessarily affected by the boundary, and so it may be argued that the navigating agent indirectly received information about the physical boundary. However, that alone is not sufficient for idiothetic localization. For example, the trajectories used to simulate iPI and aPI in Fig 1A and 1B were all affected by the kite arena (1-fold rotational symmetry), but without leading to successful localization.

The random trajectory algorithm was based on [17] which was developed to ensure that there was no clear bias in the spatial sampling by the agent, particularly near the boundary. This was important since thigmotaxis was known from pilot simulations to significantly improve localization. Empirically, there are alternative algorithms which produce nearly uniform sampling of many arenas, despite being completely agnostic of boundary contact. For instance,

If RandomNumber > 0.5

$$\tau \sim \mathrm{WN}\left(0, \sigma_\tau^2\right)$$

$$\Delta\theta_t = \Delta\theta_t + \tau\left(1.1\right)^k$$

Else

$$\tau \sim \mathrm{WN}\left(0, 25\sigma_\tau^2\right)$$

$$\Delta\theta_t = \Delta\theta_t + \tau\left(1.1\right)^k$$

End

produces a spatial sampling distribution which is approximately uniform, albeit less so than the original random trajectory algorithm which used boundary sensing information (67% vs 43% of 16-minute trajectories in a circular arena yielded a radial position distribution indistinguishable from a uniform distribution, i.e., Kolmogorov-Smirnov tests where $p>0.05$, n=1,000). Similar to the thigmotactic trajectory algorithm described earlier, the latter random trajectory algorithm does not require direct sensing of the boundary, and may be used if both the localization and trajectory generation parts of the navigation system are required to have no boundary sensing information.

Arenas

All arenas were scaled to preserve equal traversable area unless otherwise specified. The standard traversable area was equal to a circular arena of 76cm diameter, a commonly used arena for rodent experiments. Briefly, the shapes of the arenas used are described below.

- Kite – constructed by taking a 2:1 rectangle, split along the diagonal, reflecting one right triangular region, and re-joining the resulting pair of mirror-symmetric right triangular regions along the diagonal. A kite-shaped arena with two right angles is produced.
- *T-maze* – constructed by taking 5 equal square regions, concatenating 3 along the top, and 2 more down the midline of a T shape.
- *Egg* – equation from http://www16.ocn.ne.jp/~akiko-y/Egg/index_egg_E.html

$$y = \pm\sqrt{x\frac{(a-b)-2x+\sqrt{4bx+(a-b)^2}}{2}} \tag{S1}$$

where $a \geq b \geq 0$, and the long axis is the X-axis. The parameter ratios used were $b/a = 0.9$ (Fig. 2C) and $b/a = 0.7$ (Fig. 4A). The latter resulted in a more elliptic egg shape, and was used to make idiothetic localization using a standard random trajectory more difficult to clearly illustrate the improvement in localization performance due to thigmotaxis.

- *Void landmark* – a 14cm diameter circular void was centred 19cm from the circular arena centre.
- *Asymmetric barrier* – extended 25cm radially from centre of a standard circular arena.
- A 3:4:5 Pythagorean right-angled triangular arena was also used in Fig. 3B.

When present, interior boundaries/barriers were also assumed to be in memory along with the outer arena boundary.

Particle filter pose distribution update using idiothetic path integration (iPI) only

The way in which the pose distribution was updated after each step is described, taking into account the uncertainty in the size and direction of the step.

At point (x, y, θ) in pose space, the small volume $dxdyd\theta$ ideally contains $n(x, y, \theta) = Np(x, y, \theta)dxdyd\theta$ points, where N is the total number of particles, p is the true pose density function. In all particle filter simulations, $N = 10^4$, which was found in earlier work to be adequate in representing dynamic pose distributions in which idiothetic self-motion, boundary memory and boundary contacts were combined near-optimally in similar arenas [17] .

The probability density function of the true egocentric displacement $(\Delta\theta_t, \Delta r_t)$ given the estimate $(\widehat{\Delta\theta_t}, \widehat{\Delta r_t})$ was modelled as

$$f_{egomotion}\left(\Delta\theta_t, \Delta r_t \mid \widehat{\Delta\theta_t}, \widehat{\Delta r_t}\right) = \frac{1}{2\pi\sigma_0\sigma_r} e^{-\left(\frac{\left(\Delta\theta_t - \widehat{\Delta\theta_t}\right)^2}{2\sigma_0^2} + \frac{\left(\Delta r_t - \widehat{\Delta r_t}\right)^2}{2\sigma_r^2}\right)} \quad \text{(S2)}$$

where σ_0 was estimated from the drift in HD cell tuning curves in darkness, and σ_r was defined based on an estimated mean step length in darkness of 7cm, and using a liberal coefficient of variation of 0.2 [17] . The mean forward speed for simulated foraging using idiothetic cues was 9cm/s. Importantly, the model assumed that the rotational error σ_0 was constant for the duration of all simulations, and that the modelled HD drift was independent of (hence received no feedback from) the particle filter. For convenience, angular parameters of (S2) and subsequent density functions were defined over the interval $(-\infty, +\infty)$, where all angles modulo 2π denote the same physical direction.

Following each step, the exact pose distribution in allocentric pose coordinates was given by:

$$p(x_{t+1}, y_{t+1}, \theta_{t+1}) = \int_x \int_y \int_\theta f_{displace}\left(x_{t+1}, y_{t+1}, \theta_{t+1} \mid x_t, y_t, \theta_t, \widehat{\Delta\theta_t}, \widehat{\Delta r_t}\right) d\theta dy dx \quad \text{(S3)}$$

where

$$f_{displace}\left(x_{t+1}, y_{t+1}, \theta_{t+1} \mid x_t, y_t, \theta_t, \widehat{\Delta\theta_t}, \widehat{\Delta r_t}\right) = \frac{\sum_{j=-\infty}^{+\infty} e^{-\left(\frac{\sqrt{(x_{t+1}-x_t)^2+(y_{t+1}-y_t)^2}-\widehat{\Delta r_t}}{\sigma_r\sqrt{2}}\right)^2 - \left(\frac{\text{atan*}(y_{t+1}-y_t, x_{t+1}-x_t)-\left(\theta_t+\widehat{\Delta\theta_t}\right)+2j\pi}{\sigma_0\sqrt{2}}\right)^2}}{2\pi\sigma_r\sigma_0\sqrt{(x_{t+1}-x_t)^2+(y_{t+1}-y_t)^2}} \quad \text{(S4)}$$

where $\text{atan*}(y_{t+1} - y_t, x_{t+1} - x_t)$ denotes the 4-quadrant arctangent function. In the particle filter model, the egocentric displacement of each particle was drawn randomly from $f_{egomotion}\left(\Delta\theta_t, \Delta r_t \mid \widehat{\Delta\theta_t}, \widehat{\Delta r_t}\right)$, replacing the explicit calculations of the triple integral of (S3).

Particle filter pose distribution update using idiothetic self-motion cues and boundary memory

In the particle filter model, particles which crossed a boundary were culled, and remaining particles were randomly cloned to maintain a constant particle population. Importantly, the simulated agent did not have information about boundary contacts. For example, sharp turns were statistically more likely to be due to random turns within the arena than due to boundary contact (which it was unaware of).

Some possible displacements may cross a boundary according to the map in memory. Using the boundary map,

$$p\left(x_{t+1}, y_{t+1}, \theta_{t+1}\right) = \eta \int_x \int_y \int_\theta f_{displace}\left(x_{t+1}, y_{t+1}, \theta_{t+1} \mid x_t, y_t, \theta_t, \widehat{\Delta\theta_t}, \widehat{\Delta r_t}, map\right) d\theta dy dx \qquad \text{(S5)}$$

where

$$f_{displace}\left(x_{t+1}, y_{t+1}, \theta_{t+1} \mid x_t, y_t, \theta_t, \widehat{\Delta\theta_t}, \widehat{\Delta r_t}, map\right) = \begin{cases} 0 & \textit{if boundary crossed} \\ f_{displace}\left(x_{t+1}, y_{t+1}, \theta_{t+1} \mid x_t, y_t, \theta_t, \widehat{\Delta\theta_t}, \widehat{\Delta r_t}\right) & \textit{otherwise} \end{cases}$$

(S6)

where η was a normalizing constant which ensured

$$\int_x \int_y \int_\theta p\left(x_{t+1}, y_{t+1}, \theta_{t+1}\right) d\theta dy dx = 1 \qquad \text{(S7)}$$

For simulating an animal of finite size (Fig. 4A), a hypothesized pose was deemed to have crossed a boundary if any part of the animal perimeter crossed the boundary. In the majority of arenas tested, crossing a boundary nearly always equated to exiting an arena. Frequently, a test for boundary exit could therefore replace a test for boundary crossing with little or no effect on performance. However, this approximation did not hold when the arena asymmetry was due to a zero-thickness asymmetric interior barrier (Fig. 3C).

Particle filter pose distribution update using idiothetic self-motion cues, boundary memory and boundary contact information

To fuse idiothetic self-motion, boundary memory and boundary contact information (Fig. S2A), it was assumed that an estimated egocentric boundary contact vector $\left(\widehat{\phi_t}, \widehat{z_t}\right)$ was available when collision with a boundary occurred (*k>0* in the trajectory algorithm). $\widehat{\phi_t}$ was the estimated direction of the nearest boundary point in an egocentric reference frame, and $\widehat{z_t}$ was the estimated distance. Note that although boundary contact is an allothetic cue, it did not provide precise pose information because the boundary was assumed to be featureless so that any one point was indistinguishable from any other. In general, there is an infinite number of poses which exactly match a given $\left(\widehat{\phi_t}, \widehat{z_t}\right)$. For example, knowing that the boundary is precisely 5cm to the left means that the animal is somewhere along a line 5cm inside the boundary, without precise knowledge of where along a continuum of poses it may be.

The conditional probability density function of the true boundary contact vector $\left(\phi_t, z_t\right)$ given an estimate $\left(\widehat{\phi_t}, \widehat{z_t}\right)$ was modelled as

$$f_{boundary}\left(\phi_t, z_t \mid \widehat{\phi_t}, \widehat{z_t}\right) = f_{boundary}\left(\widehat{\phi_t}, \widehat{z_t} \mid \phi_t, z_t\right) = \frac{1}{2\pi\sigma_\phi\sigma_z} e^{-\left(\frac{\left(\phi_t - \widehat{\phi_t}\right)^2}{2\sigma_\phi^2} + \frac{\left(z_t - \widehat{z_t}\right)^2}{2\sigma_z^2}\right)} \qquad \text{(S8)}$$

where $\sigma_z = \sigma_r$ and $\sigma_\phi = \sigma_0$ defined the error magnitudes of boundary contact information.

The pose distribution $p\left(x_{t+1}, y_{t+1}, \theta_{t+1}\right)$ updated earlier using idiothetic self-motion and boundary memory information was refined further when boundary contact occurred (e.g., a form of allothetic localization in darkness, see Fig S2a and Ref [17]). The Bayes-optimal pose distribution given boundary contact information is given by

$$p\left(x_{t+1}, y_{t+1}, \theta_{t+1} \mid \widehat{\phi_{t+1}}, \widehat{z_{t+1}}\right) = \eta p\left(\widehat{\phi_{t+1}}, \widehat{z_{t+1}} \mid x_{t+1}, y_{t+1}, \theta_{t+1}\right) p\left(x_{t+1}, y_{t+1}, \theta_{t+1}\right) \qquad \text{(S9)}$$

where η denotes the normalizing constant, $p\left(\widehat{\phi_{t+1}}, \widehat{z_{t+1}} \mid x_{t+1}, y_{t+1}, \theta_{t+1}\right) = f_{boundary}\left(\widehat{\phi_{t+1}}, \widehat{z_{t+1}} \mid \phi_{t+1}, z_{t+1}\right)$, z_{t+1} is the shortest distance between $\left(x_{t+1}, y_{t+1}\right)$ to any boundary point, and ϕ_t is the angle measured from θ_{t+1} to that closest boundary point. In the particle filter model, each particle was assigned a weight $w = f_{boundary}\left(\widehat{\phi_{t+1}}, \widehat{z_{t+1}} \mid \phi_{t+1}, z_{t+1}\right)$ when boundary contact occurred. The weighted particles were resampled using stochastic universal resampling, resulting in a posterior distribution of unweighted particles, used as the next step's prior pose distribution.

Retrospective localization

There are multiple Bayes-optimal strategies in which localization using self-motion and boundary memory can be improved retrospectively [49] . Two strategies are demonstrated using a particle filter implementation – the first is an 'offline' reverse replay strategy, and the second is an 'online' backward inference strategy. By using memory, both strategies can be considered as passing information backward in time to improve an earlier estimate of pose. Their principles are explained using their particle filter implementations, assuming that the aim is to improve the estimate of pose at time *0*. In the first strategy (e.g., Fig 5, Fig S5), the final particle cloud at time *t* is treated as the initial pose estimate. Then the self-motion cues are replayed in reverse as input to the standard particle filter, running from *t* to *0*. Assuming that localization improved between time *0* and *t*, then the pose estimate at time *0* also improves following reverse replay. A

disadvantage of this strategy is that the sequence of self-motion cues has to be stored first (from time *0* to *t*). An advantage is that any pose estimate between *0* and *t* can be recovered during reverse replay.

In the second strategy (e.g., Fig S5), each particle is associated with two poses: the current pose (which is updated as per the standard particle filter) and the original pose at time *0* (which is not updated). Assuming that localization improved between time *0* and *t*, the total number of unique poses corresponding to time *0* will be reduced, since some particles will have no descendants. In this way, the pose estimate at time *0* can be recovered from the time *0* pose record of the descendant particles at time *t*. A disadvantage of the second strategy is that only a separately stored pose snapshot can be updated (e.g., time *0*), and which has to be decided prior to the availability of subsequent localizing information. An advantage of the second strategy is that updating is done 'online' without the need to store a sequence of self-motion cues. Alternatively, if the sequence of self-motion cues is stored (as per reverse replay), then a 'forward replay' of the sequence can begin at an arbitrary time, still using backward inference to retrospectively recover its pose. This alternative implementation avoids the need to decide the time for which to recover the pose prior to all information being available.

These two replay strategies may be considered as examples of 'beta recursions' and 'gamma recursions' respectively (for formal mathematical descriptions, see [49]). The two procedures yielded similar performance results (Fig S5, Text S1 – Supporting Results).

Continuous and intermittent compass use

The effect of using an allothetic compass continuously during path integration only (aPI, Fig. 1), and using an allothetic compass intermittently with a boundary map (stochastically, on average once every 30s, Fig. S4) were included for comparison. In both cases, the allocentric direction estimation error was assumed to be an unbiased Gaussian random variable, with $\sigma_{compass} = \sigma_0$.

Place stability index, I_p

The place stability index, I_p, was defined to measure a positional distribution's instantaneous accuracy and precision in its estimate of true position inside a bounded 2D arena [17] .

$$0 < I_P(x,y) = \frac{\langle D_0^{\,2} \mid x,y \rangle}{\langle D_0^{\,2} \mid x,y \rangle + \langle D_p^{\,2} \mid x,y \rangle} \le 1 \qquad \text{(S10)}$$

where (x,y) denotes the true position, $\langle D_0{}^2 \mid x,y \rangle$ denotes the expected squared distance of a uniform distribution of points in the arena to the true position, and $\langle D_p{}^2 \mid x,y \rangle$ denotes the expected squared distance of the distributed estimate to the true position. Applied to a particle filter, $\langle D_p{}^2 \mid x,y \rangle$ denotes the mean squared distance between particle positions and the true position. The null measure $\langle D_0{}^2 \mid x,y \rangle$ was defined based on information theoretic principles. It is analogous to the baseline uniform distribution used to define the spatial information content of place fields [20,50] .

In arenas with *n*-fold rotational symmetry, the corresponding I_p value tended to be low since even without noise, there were *n* true poses which could not be distinguished, having identical corresponding boundary representations. An adjusted I_p* value was therefore defined using *n* rotational clones of the true pose. Both $\langle D_0{}^2 \mid x,y \rangle$ and $\langle D_p{}^2 \mid x,y \rangle$ were then calculated based on the smallest distance squared to any pose clone.

Distributions of I_p values following 48 minutes, denoted by $I_p(48)$, or following 40 minutes, denoted by $I_p(40)$, used bin widths of 0.05 I_p units.

Circular variance in pose direction estimate, V(θ)

The instantaneous direction of the particle cloud was calculated as the circular mean of the particle directions, i.e.,

$$\hat{\theta} = \text{atan*}\left(\sum_j \sin(\alpha_j), \sum_j \cos(\alpha_j) \right) \qquad \text{(S11)}$$

where α_j denotes the allocentric direction of particle *j*, and $\text{atan*}(y,x)$ denotes the 4-quadrant arctangent function. Across *n* trials, the circular variance [51] of the error in pose direction estimate was defined as

$$0 \le V(\theta) = 1 - \left(\overline{\cos\left(\widehat{\theta_k} - \theta_k^{TRUE}\right)} \right)^2 - \left(\overline{\sin\left(\widehat{\theta_k} - \theta_k^{TRUE}\right)} \right)^2 \le 1 \qquad \text{(S12)}$$

where $\widehat{\theta_k}$ was the instantaneous directional estimate of the particle cloud of trial *k* found using (S11), and θ_k^{TRUE} was the instantaneous true direction of trial *k*. Note that the circular variance is independent of net directional bias and cannot be used to quantify the instantaneous net deviation of a single particle filter's direction estimate from the true direction. However, across all random simulation trials, the net deviations were unbiased and the circular variance therefore reflected the magnitude of directional error. Consequently,

$V(\theta)$ is a measure of the spread of best direction estimates around the true direction across trials, rather than a measure of an individual particle filter's directional accuracy or precision.

Grid cell spike simulation

A simple spiking neuron simulation was used to test whether the particle filter's pose estimate was capable of maintaining a stable representation of position similar to the firing fields of cells in the mammalian hippocampal-entorhinal space circuit. To maintain a multimodal grid field requires a higher level of spatial specificity than a unimodal place field. The finest grid spacing observed *in vivo* is approximately 30cm [13,18] , which would be the most difficult grid to resolve spatially in the presence of positional uncertainty. This was chosen as the simulated grid spacing to test localization performance.

Spike probability following each step was modelled as

$$p_j = e^{-r_j^2/2\sigma_{grid}^2} \qquad \text{(S13)}$$

where $r_j^2 = \left(x_{f_j} - \overline{x_t}\right)^2 + \left(y_{f_j} - \overline{y_t}\right)^2$ and $\sigma_{grid} = 2.5\,\text{cm}$ for all standard arenas, and $\sigma_{grid} = 5\,\text{cm}$ for simulations using 2-fold standard grid spacing (Fig S3B, S4B). The spike probability decreased monotonically from unity according to the distance r_j between the positional centre of mass of the particle cloud $\left(\overline{x_t}, \overline{y_t}\right)$ and ideal firing position $\left(x_{f_j}, y_{f_j}\right)$ of grid mode *j*. Since $\sigma_{grid} << 30cm$, in practice at most one ideal firing position contributed significantly to spike generation at any moment. Ideal firing positions were defined explicitly to be at the grid points of a tessellating hexagonal grid. The phase and orientation of ideal grid points were empirically adjusted to include at least three points inside each arena. Ideal adjacent grid positions were 30cm apart for all arenas except the enlarged arenas of Fig S3B and S4B where a 60cm grid spacing was used. The simulated spikes from all trials were pooled to provide a single representation of the spatial specificity which could be expected over many random trajectories within an arena.

A gridness index (Table S1) was found using the autocorrelogram of the firing rate map [19] . Where grid modes were indistinct, a circular annulus mask was used with inner radius 0.5x, and outer radius 1.5x the ideal grid spacing.

The spikes corresponding to the three modes with the highest total spike count were further analysed using methods developed for place fields (Table S1). The maximum likelihood factorial model [20] was used to find a mode's spatial and direction information content. This analysis provided a measure of the time-averaged spatial specificity achieved in three different locations of each arena, correcting for possible direction-dependence. The standard spike and position histogram bin sizes used were 2.5cm x 2.5cm for

position and 6° for direction. For simulations using 2-fold standard grid spacing, bin sizes of 5 cm x 5 cm x 6° were used.

Test of point density uniformity in an arena

Test of point density uniformity (Fig S3B) was performed by taking the voronoi mesh polygons (of spike positions) which were completely within the arena boundary, and comparing their area distribution against an equivalent area distribution obtained from an equal-size random sample of points uniformly distributed in the same arena (Kolmogorov-Smirnov test).

Test for equal circular concentrations

The κ-test is a parametric two-sample test to determine whether two circular concentration parameters are different, assuming the direction samples arise from von Mises distirbutions [51] . The test was implemented as *circ_ktest* in the Circular Statistics toolbox for Matlab [52] .

Kinetic time constant

The duration t_{90} was defined as the time (minutes:seconds) taken for the median I_p to undergo 90% of the total increase (or decrease) over 48 minutes.

Arena rotational asymmetry

The rotational symmetry of an arena was determined by rotating through 360°. If a rotated arena is identical to the unrotated arena only once through the rotation, i.e., at 360° (≡0°), then it has 1-fold rotational symmetry (abbreviated as 1-RS here). For example, ellipses and rectangles have two rotation angles (180° and 360°) where they are identical to the unrotated arena, hence have 2-fold rotational symmetry (2-RS).

An arena rotational asymmetry function (Fig. 3B, left) was found by rotating an arena around its centre of mass, overlaid on the original arena. Asymmetry was calculated as the normalized non-overlapping area (between 0 and 1). In arenas with n-RS (n>1), there are n zero-points in the asymmetry function. The mean asymmetry over 360° was used in Fig 2b (right). It should be noted that this function did not account for trajectory-dependent inhomogeneity in arena sampling, or crossing of interior boundaries (Fig. 3C).

Supporting Results

Effects of matched and mismatched noise magnitude on idiothetic localization

Increasing either the magnitude of angular (Fig. S1A) or linear (Fig S1B) displacement estimation noise resulted in a significant deterioration in idiothetic localization performance. $\widetilde{I_P}$ values were inversely related to the magnitude of both types of noise (Fig. S1E). These results occurred despite the navigation system matching the true noise distribution during particle filter update, showing that the effects on performance were directly attributable to the level of noise in the estimate of self-motion. Under all 'matched noise' conditions, >91% of I_P values remained above chance after 48 minutes. Similarly, >76% of $\theta = \hat{\theta} - \theta^{TRUE}$ (error in pose direction estimate) remained within ±45° of the true heading. These results are consistent with the fact that even at the highest levels of matched linear or angular noise, individual grid modes showed moderate to high spatial information content (>1.2 bits/spike, Table S1 – S1A, S1B). Therefore, although a multimodal firing field may not be evident (gridness indices <0.3), unimodal firing patterns similar to that of place fields may still be possible under these conditions.

Next, the magnitude of angular or linear noise was under- or over- estimated by the navigation system, while maintaining the same baseline level of true angular and linear noise (Fig. S1C, S1D). Under all 'mismatched noise' conditions, >85% of I_P values remained above chance after 48 minutes. Similarly, >75% of θ remained within ±45° of the true heading. Large underestimation of angular or linear noise (0.25x, light green) resulted in a rise in the secondary mode of the distribution of $I_P(48)$, caused by the presence of gross localization errors in a small proportion of trials. This was associated with an increase in $V(\theta)$, which in the case of angular noise, actually increased over 48 minutes (Fig S1C light green). Over time, underestimation of angular noise can therefore cause deterioration in idiothetic localization performance, despite successful initial localization. In contrast, overestimation of errors reduced $\widetilde{I_P}$ but resulted in a smaller $V(\theta)$. The latter may be attributed to the reduction of gross localization errors which sporadically occurred due to the use of a finite particle cloud population to model a continuous distribution. Overall, the results suggest that underestimation of self-motion noise increases the frequency of large idiothetic localization errors. The relatively high spatial information content (>1.6 bits/spike) and relatively low directional information content (<0.2 bits/spike) of individual grid modes, together with the gridness index (>0.25), show that both unimodal and multimodal firing patterns can be sustained despite mismatched self-motion noise (Table S1 – S1C, S1D).

Taken together, these results show that while the magnitude of angular and linear noise affected localization performance, idiothetic localization is still possible despite a mismatch between the true noise parameters and that used by the navigation system.

Intermittent use of boundary contact information

During periods with additional boundary contact information (Fig. S2A & S2B, yellow bars), $\widetilde{I_P}$ increased and $V(\theta)$ decreased. Conversely, following cessation of use of boundary contact information (Fig. S2A & S2B, orange bars), $\widetilde{I_P}$ decreased and $V(\theta)$ increased. Having initial pose information (oriented) was associated with a small but statistically significant residual increase in $\widetilde{I_P}$ at 40 and 48 minutes, with and without intervening periods of using boundary contact information (Fig. S2A, right). In contrast, the difference in $\widetilde{I_P}(40)$ was not statistically significant (Bonferroni correction for family-wise type I error), comparing with or without a history of two 8-minute periods of using boundary contact information. These results suggest that quality of initial pose information may have a greater residual effect on localization performance than intermittent allothetic boundary contact information. Commensurate changes in properties of simulated firing patterns were evident (Fig. S2B, Table S1).

Effects of arena size on idiothetic localization

A 4-fold increase in the kite arena area (Fig. S3A) resulted in a lower $\widetilde{I_P}$ (right, Wilcoxon test, $p=1.4\text{x}10^{-12}$) and higher $V(\theta)$ (κ-test, $p<10^{-16}$) at 48 minutes, showing that localization performance was affected by arena area. There was a commensurate decrease in the spatial specificity of simulated grids. At the standard grid scale, no grid modes were detected (Fig. S3B, left, spike uniformity test, p=0.60). This was partially rescued by doubling the grid spacing (Fig. S3B, 2nd from left, spike uniformity test, $p<10^{-16}$). The crosscorrelogram between the grid fields of the standard and scaled large arena (Fig. S3B, right) showed the overall grid pattern was preserved despite a less distinct grid in the large arena. These results suggest the boundary's effect on idiothetic localization is reduced in large arenas, and performance may be poor.

A comparison of two retrospective localization procedures

Two retrospective localization procedures were implemented using the particle filter model described earlier. One was an 'offline' reverse replay procedure, the other an 'online' backward inference procedure, sharing the common feature that future self-motion information is used retrospectively to improve the

estimate of pose (detailed in Text S1 – Modelling and Analysis). In a comparison study (Fig S5), both procedures were used to obtain the pose estimate at time *0* using the same environment and assumptions as Fig 5. Due to the large number of particles culled during 'online' backward inference, 10^6 particles were used for all simulations in this comparison. Starting from full disorientation, both procedures yielded a significant retrospective increase in $\widetilde{I_P}$ at time *0* (Wilcoxon signed rank test, $p<10^{-100}$ for both procedures, $n = 10^3$), demonstrating that both procedures successfully utilized future self-motion information to improve a past pose estimate. The $\widetilde{I_P}$ using 'offline' reverse replay was marginally higher than 'online' backward inference (0.89 and 0.88 respectively, Wilcoxon rank sum test, p=0.003). However, the error distances between true position and estimated position at time *0* were not significantly different between the two procedures (mean±s.d., 'offline' reverse replay: 12.7±12.3 cm, 'online' backward inference: 12.8±11.2 cm, Wilcoxon rank sum test, p=0.12, *t*-test, p=0.76). Taken together, these results demonstrate that at least two distinct particle filter implementations of retrospective localization can be used to recover a pose from whence a navigating agent was fully disoriented, and without using allothetic cues.

Supporting References

49. Penny WD, Zeidman P, Burgess N (2103) Forward and backward inference in spatial cognition. PLoS Comput Biol 9: e1003383.

50. Skaggs WE, McNaughton BL, Gothard KM, Markus EJ. An information-theoretic approach to deciphering the hippocampal code. In: Hanson SJ, Cowan JD, Gilles CL, editors; 1993; San Mateo, CA, USA. Morgan Kaufmann Publishers. pp. 1030-1037.

51. Batschelet E (1981) Circular statistics in biology. London; New York: Academic Press. xvi, 371 pp.

52. Berens P (2009) CircStat: A MATLAB Toolbox for Circular Statistics. J Stat Softw 31: 1-21.
